# Evaluation and Comparison of the Efficiency of Transcription Terminators in Different Cyanobacterial Species

**DOI:** 10.3389/fmicb.2020.624011

**Published:** 2021-01-15

**Authors:** Grant A. R. Gale, Baojun Wang, Alistair J. McCormick

**Affiliations:** ^1^School of Biological Sciences, Institute of Molecular Plant Sciences, University of Edinburgh, Edinburgh, United Kingdom; ^2^Centre for Synthetic and Systems Biology, University of Edinburgh, Edinburgh, United Kingdom; ^3^School of Biological Sciences, Institute of Quantitative Biology, Biochemistry and Biotechnology, University of Edinburgh, Edinburgh, United Kingdom

**Keywords:** CyanoGate, *Escherichia coli*, Golden Gate, intrinsic terminator, MoClo, *Synechococcus elongatus* UTEX 2973, *Synechocystis* sp. PCC 6803, synthetic biology

## Abstract

Cyanobacteria utilize sunlight to convert carbon dioxide into a wide variety of secondary metabolites and show great potential for green biotechnology applications. Although cyanobacterial synthetic biology is less mature than for other heterotrophic model organisms, there are now a range of molecular tools available to modulate and control gene expression. One area of gene regulation that still lags behind other model organisms is the modulation of gene transcription, particularly transcription termination. A vast number of intrinsic transcription terminators are now available in heterotrophs, but only a small number have been investigated in cyanobacteria. As artificial gene expression systems become larger and more complex, with short stretches of DNA harboring strong promoters and multiple gene expression cassettes, the need to stop transcription efficiently and insulate downstream regions from unwanted interference is becoming more important. In this study, we adapted a dual reporter tool for use with the CyanoGate MoClo Assembly system that can quantify and compare the efficiency of terminator sequences within and between different species. We characterized 34 intrinsic terminators in *Escherichia coli*, *Synechocystis* sp. PCC 6803, and *Synechococcus elongatus* UTEX 2973 and observed significant differences in termination efficiencies. However, we also identified five terminators with termination efficiencies of >96% in all three species, indicating that some terminators can behave consistently in both heterotrophic species and cyanobacteria.

## Introduction

Cyanobacteria comprises a large and diverse phylum of photoautotrophic bacteria that can capture and convert inorganic carbon (e.g., CO_2_) into a wide variety of secondary metabolites (Huang and Zimba, 2019). Many cyanobacterial species are genetically tractable and show great potential for green biotechnology applications, such as the sustainable production of biofuels and high value biomolecules (Lin et al., 2017; Knoot et al., 2018; Eungrasamee et al., 2019; Lin and Pakrasi, 2019; Włodarczyk et al., 2019). Much of the recent progress in engineering cyanobacteria has been driven by the uptake of synthetic biology approaches. One major aim of cyanobacterial synthetic biology is the development of new tools and strategies to facilitate stringent and precise control of gene expression. A wide variety of new molecular tools and genetic parts to tune gene expression are now available for use by the research community (Englund et al., 2016; Kim et al., 2017; Ferreira et al., 2018; Kelly et al., 2018; Vasudevan et al., 2019; Yao et al., 2020). The increase in availability of well-characterized genetic parts has allowed rational design, a core process to the synthetic biology paradigm, to be more routinely employed in the engineering of new cyanobacterial strains. Nevertheless, the majority of synthetic biology work in cyanobacteria has thus far concentrated on characterizing genetic elements that control gene transcription (e.g., promoters, CRISPRi) or translation modulation (e.g., ribosomal binding sites (RBS), riboswitches, small RNAs) (Huang and Lindblad, 2013; Camsund et al., 2014; Ma et al., 2014; Immethun et al., 2017; Kelly et al., 2018; Sun et al., 2018; Behle et al., 2020; Yao et al., 2020). Transcription terminators are also key transcriptional control elements, but far fewer studies have examined their roles in regulating gene expression in cyanobacteria.

The rational design of efficient gene expression cassettes (and more advanced gene circuits) requires the use of genetic parts with well-characterized and predictable function (Moser et al., 2018). For instance, strong terminators attenuate transcription and isolate downstream genetic sequences, which can prevent interference and disruption of function from unwanted transcriptional readthrough (Kelly et al., 2019). This is particularly important when considering synthetic gene constructs, where several gene expression cassettes driven by strong promoters may occupy a short stretch of DNA. Furthermore, many prokaryotes (including cyanobacteria) are prone to homologous recombination. Homologous regions as small as 23–27 bp have been demonstrated to lead to recombination in *Escherichia coli*, so multiple distinct terminators are generally preferable for multi-gene expression systems and gene circuits (Shen and Huang, 1986; Sleight et al., 2010; Chen et al., 2013). As with other genetic parts, an understanding of terminator performance and robustness between species is also important. Promoters have been shown to drive gene expression differently in cyanobacteria compared to heterotrophic species (e.g., *Escherichia coli*) and between cyanobacterial species (Camsund et al., 2014; Vasudevan et al., 2019). In contrast, potential differences in behavior between cyanobacterial species has not yet been investigated for transcription terminators.

In prokaryotes, transcription is terminated by two distinct terminator types: (i) Rho-dependent terminators that rely on a Rho transcription factor, and (ii) Rho-independent, or intrinsic terminators, which do not require a transcription factor. In *E. coli*, approximately 20% of terminators are Rho-dependent (Peters et al., 2009). However, Rho transcription factors appear to be absent in cyanobacteria, such that all transcription termination events are thought to rely on intrinsic termination (Vijayan et al., 2011). Intrinsic terminators are defined by a sequence motif that forms a hairpin loop secondary structure in the nascent RNA transcript. The hairpin loop is comprised of a GC-rich stem (8–12 nucleotides) (nt) and a loop (3–6 nt). Upstream of the hairpin loop is an adenine-rich region (the A-tract) typically 6–8 nt in length, while downstream is a uracil-rich region of 7–12 nt in length (the U-tract). Intrinsic termination depends upon the differential binding affinities between nucleotides. The interaction between U and A is weak, such that transcription of the U-tract results in a pause in transcription that allows the hairpin loop to form. The presence of the hairpin loop in the RNA polymerase (RNAP) exit channel, causes a ratcheting action and subsequent disruption of RNA-DNA binding. This leads to dissociation of RNAP from the DNA template and the subsequent release of the nascent RNA transcript (Wilson and Von Hippel, 1995; Herbert et al., 2008; Peters et al., 2011). In *E. coli*, many terminators have been assessed for termination efficiency (TE), which is typically calculated as a percentage estimate of the RNAP transcription elongation complexes prevented from continuing transcription passed a given sequence (i.e., a terminator) (Cambray et al., 2013; Chen et al., 2013). Importantly, a “no terminator” control was included to determine a normalized value for TE in those studies.

Characterization studies of terminators in cyanobacteria are currently limited to the model species *Synechocystis* sp. PCC 6803 (PCC 6803). Liu and Pakrasi (2018) evaluated the relative strengths of seven native terminators using a dual fluorescent reporter system similar to that used by Chen et al. (2013). More recently, Kelly et al. (2019) evaluated 19 synthetic and heterologous intrinsic terminators ported from *E. coli*, with the aim of identifying terminators able to insulate a specific genomic locus in PCC 6803 from native promoter readthrough originating from upstream of the insertion site. Each terminator sequence was inserted between the transcription start site (TSS) and RBS of an inducible promoter driving *YFP*, and following induction, twelve terminators were shown to efficiently block transcription indicating a potential efficiency of nearly100%. These studies have provided valuable insights into terminator function in PCC 6803. But if comparisons in performance between different strains are to be achieved, a normalized quantitative parameter, such as TE, should be calculated.

In this study we assembled a set of 34 intrinsic terminators from PCC 6803, and *E. coli* and synthetic libraries that have previously demonstrated a wide range of TE values in *E. coli* (Chen et al., 2013). We re-designed an established dual fluorescent reporter system to be compatible with the CyanoGate MoClo Assembly system, which allowed for increased cloning throughput (Liu and Pakrasi, 2018; Vasudevan et al., 2019). Importantly, all assays included a “no terminator” control vector as a reference to calculate a normalized TE value for each terminator, such that the TE values could be compared between different experiments and species irrespective of the instrument or gain settings used. We first validated and benchmarked our testing system by comparing TE values from the literature with our results in *E. coli*. Then we tested the performance of the terminators in two different cyanobacterial species: PCC 6803 and the recently described high-light tolerant *Synechococcus elongatus* UTEX 2973 (UTEX 2973) (Williams, 1988; Yu et al., 2015).

## Materials and Methods

### Cyanobacterial Culture Conditions

The *Synechocystis* sp. PCC 6803 glucose tolerant (GT) strain (obtained from the Lea-Smith lab at the University of East-Anglia, United Kingdom) (Zavøel et al., 2017) and UTEX 2973 were maintained on 1.5% (w/v) agar plates containing BG11 medium (Lea-Smith et al., 2016). Liquid cultures were grown in BG11 (supplemented with 10 mM NaHCO_3_) in 100 ml Erlenmeyer flasks. Liquid cultures were shaken at 100 rpm and aerated with filter-sterilized, water-saturated air. PCC 6803 and UTEX 2973 transconjugants were cultured in BG11 medium and on BG11 agar plates, supplemented with 50 μg/ml kanamycin (BG11 + Kan50). Strains were grown under continuous light with PCC 6803 grown at 30°C, 100 μmol photons m^–2^ s^–1^ and UTEX 2973 at 40°C, 300 μmol photons m^–2^ s^–1^ in a Multitron Pro incubator supplied with warm white LED lighting (Infors HT).

### Vector Construction and Parts Assembly

All cloning was performed in OneShot TOP10 *E. coli* cells. Transformed cells were cultured in LB medium and on 1.5% (w/v) LB agar plates supplemented with either 100 μg/ml spectinomycin or 50 μg/ml kanamycin as required. *E. coli* strain MC1061 was cultured in LB medium supplemented with 100 μg/ml ampicillin and 25 μg/ml chloramphenicol. All *E. coli* strains were grown at 37°C with shaking at 225 rpm.

pPMQAK1-T (pCAT.000) from the CyanoGate toolkit was modified to generate pDUOTK1-L1 (pCA1.332, Addgene vector ID 162351)^1^ (Supplementary Information S1) (Vasudevan et al., 2019). To assemble pDUOTK1-L1, pPMQAK1-T was first digested with *Bpi*I and *Bsa*I (Thermo Fisher Scientific). The linearized backbone was gel purified using a Monarch DNA Gel Extraction Kit (NEB). Sequences encoding P*_*trc*__10_*-*eYFP* from the CyanoGate vector pCAT.262, the *LacZ* expression cassette from the Plant MoClo level 1 acceptor vector pICH47732 and *mTagBFP-*T_rrnB_ (from an available vector containing BBa_K592100)^2^ fused at the 5′ end to the RBS-associated sequence used by Chen et al. (2013) (BBa_B0034) were amplified using Q5 High-Fidelity DNA Polymerase (NEB) (Supplementary Table S1). Finally, the three amplicons and the linearized pPMQAK1-T backbone were assembled together using Golden Gate assembly (Vasudevan et al., 2019). pDUOTK1-L1 contains *Bsa*I restriction sites flanking *LacZ* that generate overhangs GCTT-CGCT, such that level 0 terminator parts can be assembled directly and screened using blue-white selection.

Terminator parts were generated by overlap extension PCR using two synthesized oligonucleotides (Integrated DNA Technology) (Supplementary Table S1), and the resulting amplicons were assembled into the level 0 (3U + Ter) acceptor vector pICH41276 (Supplementary Information S1) (Engler et al., 2014). New level 0 terminator parts and existing parts from CyanoGate toolkit (Addgene Kit #1000000146)^3^ were assembled into pDUOTK1-L1 to give vectors pC1.342 to pC1.375 (Supplementary Table S2).

Two “no terminator” control vectors were generated to determine 0% TE (i.e., the maximum ratio of mTagBFP relative to eYFP). pC1.376 was assembled as pDUOTK1-L1 above, but without inclusion of *LacZ* (Supplementary Information S1). For pC1.377, the spacer sequence rd1.2 (5′-cgcccccggaggctttcccggggcaaatca-3′) from Cambray et al. (2013) was generated using overlap extension PCR (Supplementary Table S1), and the PCR product was assembled into pDUOTK1-L1 using Golden Gate assembly.

### Cyanobacterial Conjugation

Genetic modification by conjugation in PCC 6803 and UTEX 2973 was facilitated by *E. coli* strain MC1061 carrying the mobilizer vector pRK24^4^ and helper vector pRL528^5^ (Tsinoremas et al., 1994; Gale et al., 2019). Conjugal transfer was performed as in Gale et al. (2019).

### Fluorescence Assays

To measure fluorescence in *E. coli*, transformants were first inoculated into 5 ml LB medium supplemented with 50 μg/ml kanamycin and grown overnight at 37°C with constant shaking at 225 rpm. To initiate the assay, overnight cultures were diluted 1:1000 into a black 96 well flat bottom plate (F-Bottom (Chimney Well) μCLEAR^®^, Greiner Bio-One) containing fresh LB medium supplemented with 50 μg/ml kanamycin to a final volume of 200 μl. The plates were incubated at 37°C with constant shaking at 600 rpm and culture density (OD_600_) was measured hourly using a FLUOstar OMEGA microplate reader (BMG Labtech). At early exponential phase (*ca*. 4.5 h following inoculation), eYFP and mTagBFP fluorescence levels were measured for individual cells by flow cytometry (minimum 10,000 cells per culture) with a FACSCanto II with HTS Flow Cytometer (Becton Dickinson). Cells were gated using forward and side scatter. Median eYFP and mTagBFP fluorescence levels were calculated from excitation/emission wavelengths 488 nm/530/30 nm and 407 nm/450/50 nm, respectively. An “empty” pPMQAK1-T vector (i.e., with no eYFP or mTagBFP expression cassettes) was included as a base line control. Fluorescence values for the latter control were subtracted from transconjugant strain measurements.

To measure fluorescence in cyanobacteria, PCC 6803 or UTEX 2973 transconjugants maintained on BG11 + Kan50 agar plates were first inoculated into 10 ml BG11 + Kan50 medium and grown for 2–3 days to OD_750_ ∼1.0. To initiate the assay, the seed cultures were diluted to a starting OD_750_ of 0.2 in 24-well plates (Costar Corning Incorporated) containing fresh BG11 + Kan50 medium to a final volume of 2 ml. Cultures were grown for three days under culturing conditions and high humidity (95%) to avoid evaporation. eYFP and mTagBFP fluorescence were measured by flow cytometry for individual cells (minimum 10,000 cells per culture) with an LSRFortessa SORP with HTS Flow Cytometer (Becton Dickinson). Cells were gated using forward and side scatter. Median eYFP and mTagBFP fluorescence levels were calculated from excitation/emission wavelengths 488 nm/515–545 nm and 407 nm/425–475 nm, respectively. As above, a base line control was included for each species.

### Calculations for Termination Efficiency

TE was calculated as a percentage from the ratio of the mTagBFP fluorescence signal downstream of the terminator to the eYFP fluorescence signal upstream relative to a control containing no terminator between fluorescent reporters:

(1) $$\Delta \,T\,e\,r\,m_{0}=\frac{B\,F\,P_{0}}{Y\,F\,P_{0}}$$

Where BFP_0_ and YFP_0_ are the mTagBFP and eYFP fluorescence signals, respectively, of the strain containing either pCA1.376 or pCA1.377.

(2) $$T\,E=100-\left(\frac{B\,F\,P_{T\,e\,r\,m}}{Y\,F\,P_{T\,e\,r\,m}}\times \frac{1}{\Delta \,T\,e\,r\,m_{0}}\times 100\right)$$

Where BFP_Term_ and YFP_Term_ are the mTagBFP and eYFP fluorescence signals, respectively, of a strain carrying a given level 1 terminator vector (Supplementary Table S2).

### Statistical Analysis

Significant differences between sample groups were assessed by one-way ANOVA followed by Tukey’s honest significant difference (HSD) *post-hoc* test using GraphPad Prism (version. 8.4.2).

### Estimation of Gibbs Free Energy

Estimated Gibbs free energy values were generated using mFold v3.0^6^ (Zuker, 2003). Free energy values were calculated without adjustment of the standard parameters, which included a fixed temperature of 37°C.

## Results

### Generating a Screening System for Level 0 Terminator Parts

**FIGURE 1:**
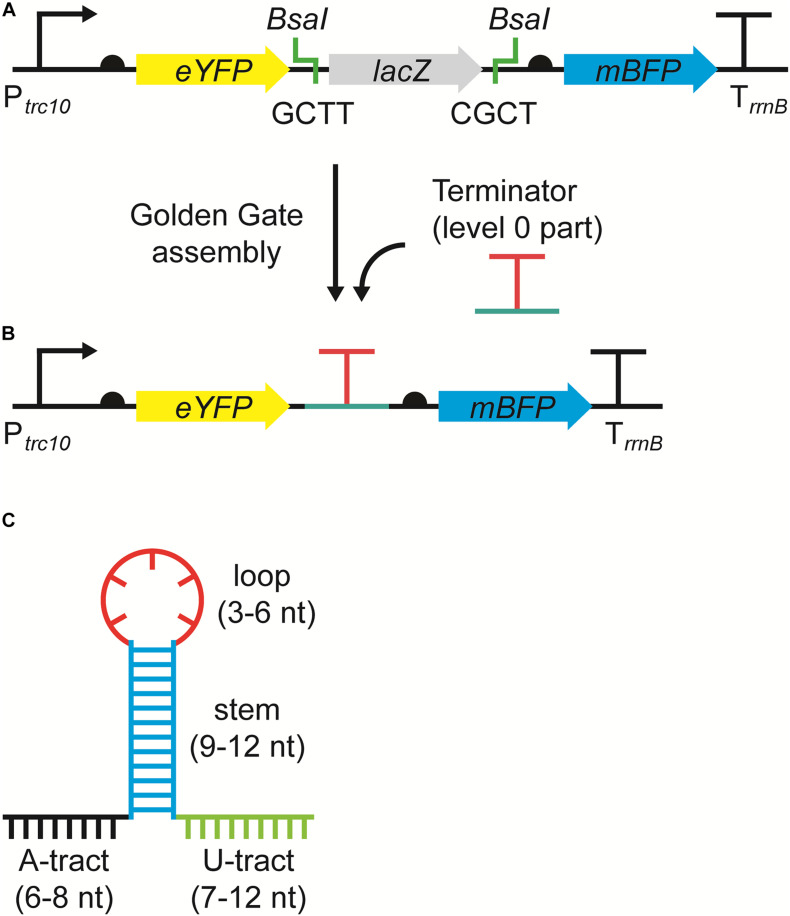
The dual fluorescence reporter system for screening terminators. **(A)** The acceptor vector pDUOTK1-L1 contains two *Bsa*I sites that generate 4 nucleotide (nt) overhangs (i.e., GCTT and CGCT) following restriction, which are compatible with standard level 0 terminator parts (Engler et al., 2014). **(B)** Following a level 1 Golden Gate assembly reaction (Vasudevan et al., 2019), the level 0 terminator part is inserted between eYFP and mTagBFP and the dual fluorescent reporter system is formed, which can then be used to evaluate termination efficiency (TE). The reporter system is driven by the strong promoter P*_*trc*__10_* and is terminated by the terminator T*_*rrnB*_*. Ribosome binding sites (half circles) are indicated (see Supplementary Information S1 for sequence details). **(C)** Example of an intrinsic terminator structure and nt sequence, comprised of an adenine rich region (A-tract) (black), followed by a G-C rich stem (blue), a hairpin loop (red), and a uracil rich region (U-tract) (green).

The RSF1010-based level T acceptor vector pPMQAK1-T from the CyanoGate toolkit was modified to generate the new level 1 acceptor vector pDUOTK1-L1 for terminator screening (Figure 1A and Supplementary Information S1) (Vasudevan et al., 2019). pDUOTK1-L1 comprises a dual fluorescent reporter system with eYFP and mTagBFP, similar to that in Liu and Pakrasi (2018). Terminators can be assembled as level 0 parts into pDUOTK1-L1 using Golden Gate assembly (Figure 1B), while the RSF1010 origin of replication allows for screening in a wide range of species (Mermet-Bouvier et al., 1993).

We compiled a library of 34 level 0 vectors containing intrinsic transcription terminators (Table 1 and Figure 1C), and then assembled these into pDUOTK1-L1 (Supplementary Table S2). In order to maximize potential orthogonality with terminators in cyanobacterial genomes, we primarily targeted heterologous terminator sequences. The library included 22 native terminators from *E. coli* and eight synthetic terminators based on *E. coli* sequences that have been previously characterized in *E. coli* (Chen et al., 2013). We also included T_rrnB_ (i.e., T_rrnB_ from *E. coli* and the T7 viral terminator in tandem (Vasudevan et al., 2019)) and the pSB1AK3 terminator (T_pSB__1A__K__3_) that was derived from the *E. coli* ribosomal RNA rrnC operon and is used in several BioBricks vectors, including pPMQAK1, to flank the cloning site (Huang et al., 2010). From PCC 6803, the terminator of the highly expressed D1 subunit of photosystem II was included (T_psbA__2_), as we expected it to have a high efficiency of termination. In contrast, T_psaB_ was included as a potentially low efficiency terminator based on previous work (Liu and Pakrasi, 2018). Two “no terminator” control vectors, pC1.376 and pC1.377, were assembled based on sequences used in previous *E. coli* studies (Cambray et al., 2013; Chen et al., 2013). In pC1.376, eYFP and mTagBFP were separated only by an RBS-associated sequence, while pCA1.377 included a spacer sequence reported to be inert (i.e., free from promoter or terminator activity in *E. coli*) (Supplementary Information S1).

**TABLE 1:** List of terminators used in this study.

**Vector ID**	**Part name**	**ΔG_*A*_ (kcal/mol)**	**Length (bp)**	**Terminator sequence**	**Origin**	**Reference**
pC0.291	T_L__3S2P__21_	–11.0	61	CTCGGTACCAAATTCCAGAAAAGAGGCCTCCCGAAAGGGGGGCCTTTTTTCGTTTT GGTCC		
pC0.292	T_L__3S2P__11_	–11.0	57	CTCGGTACCAAATTCCAGAAAAGAGACGCTTTCGAGCGTCTTTTTTCGTTTTGGTCC		
pC0.293	T_L__3S2P__55_	–2.8	57	CTCGGTACCAAAGACGAACAATAAGACGCTGAAAAGCGTCTTTTTTCGTTTTGGTCC		
pC0.294	T_L__3S3P__21_	–4.1	53	CCAATTATTGAAGGCCTCCCTAACGGGGGGCCTTTTTTTGTTTCTGGTCTCCC	Synthetic	Chen et al., 2013
pC0.295	T_L__3S1P__13_	–2.8	51	GACGAACAATAAGGCCTCCCTAACGGGGGGCCTTTTTTATTGATAACAAAA		
pC0.296	T_L__3S3P__11_	–4.2	47	CCAATTATTGAACACCCTTCGGGGTGTTTTTTTGTTTCTGGTCTCCC		
pC0.306	T_L__3S1P__22_	–2.8	48	GACGAACAATAAGGCCGCAAATCGCGGCCTTTTTTATTGATAACAAAA		
pC0.307	T_L__3S1P__47_	–8.4	52	TTTTCGAAAAAAGGCCTCCCAAATCGGGGGGCCTTTTTTTATAGCAACAAAA		

pC0.066	T_*pheA*__–__1_	–2.8	52	GACGAACAATAAGGCCTCCCAAATCGGGGGGCCTTTTTTATTGATAACAAAA		
pC0.068	T_ECK__120010850_	–4.4	45	AGTTAACCAAAAAGGGGGGATTTTATCTCCCCTTTAATTTTTCCT		
pC0.069	T_ECK__120026481_	–6.3	54	TACCACCGTCAAAAAAAACGGCGCTTTTTAGCGCCGTTTTTATTTTTCAACCTT		
pC0.072	T_ECK__120010842_	–2.5	47	CCGACGTAAAAAGACGGTAAGTATCGCTTTCAGTCTTATGAATATCG		
pC0.074	T_ECK__120048902_	–7.9	36	GCGTAAAAAAGCACCTTTTTAGGTGCTTTTTTGTGG		
pC0.062	T_Bba_B__0011_	–5.5	46	AGAGAATATAAAAAGCCAGATTATTAATCCGGCTTTTTTATTATTT	*E. coli*	Chen et al., 2013;
pC0.064	T_ECK__120010820_	–5.3	33	CTAAGCGTTGTCCCCAGTGGGGATGTGACGAAG		Vasudevan et al., 2019
pC0.070	T_Bba_B__0061_	–13.1	31	AAGTCAAAAGCCTCCGGTCGGAGGCTTTTGACTTT		
pC0.071	T_ECK__120030798_	–5.9	42	AGAATAAATTCAACCGCCCGTCAGGGCGGTTGTCATATGGAG		
pC0.073	T_ECK__120010869_	–5.6	35	TAACGTAAAAACCCGCTTCGGCGGGTTTTTTTATG		
pC0.077	T_ECK__120010841__–__*R*_	–3.0	41	AAAAACAAAAACCCCGGACTCTCATCCAGGGTTCTCTGCTT		

pC0.308	T_ECK__120033737_	–8.0	57	GGAAACACAGAAAAAAGCCCGCACCTGACAGTGCGGGCTTTTTTTTTCGACCAAAGG		
pC0.309	T_ECK__120033736_	–8.7	53	AACGCATGAGAAAGCCCCCGGAAGATCACCTTCCGGGGGCTTTTTTATTGCGC		
pC0.310	T_ECK__120010818_	–10.8	54	CACCTGTTTTACGTAAAAACCCGCTTCGGCGGGTTTTTACTTTTGG		
pC0.311	T_ECK__120015440_	–6.4	49	TCCGGCAATTAAAAAAGCGGCTAACCACGCCGCTTTTTTTACGTCTGCA		
pC0.312	T_ECK__120029600_	–4.8	90	TTCAGCCAAAAAACTTAAGACCGCCGGTCTTGTCCACTACCTTGCAGTAATGCGGTG GACAGGATCGGCGGTTTTCTTTTCTCTTCTCAA		
pC0.313	T_ECK__120010799_	–10.6	60	TCAGGAAAAAAGGCGACAGAGTAATCTGTCGCCTTTTTTCTTTGC	*E. coli*	Chen et al., 2013
pC0.314	T_ECK__120010876_	–5.6	55	GAAAAATAAAAACGGCGCTAAAAAGCGCCGTTTTTTTTGACGGT		
pC0.315	T_ECK__120015170_	–8.5	47	TTTCGAAAAAACCCGCTTCGGCGGGTTTTTTTATAGC		
pC0.316	T_ECK__120017009_	–5.5	44	GATCTAACTAAAAAGGCCGCTCTGCGGCCTTTTTTCTTTTCACT		
pC0.317	T_ECK__120051401_	–7.4	47	ATAGCAAAAAAGCGCCTTTAGGGCGCTTTTTTACATTG		
pC0.318	T_ECK__120010855_	–5.7	42	AACAACGGAAACCGGCCATTGCGCCGGTTTTTTTTGGCC		

pC0.082	T_rrnB_	–12.2	123	CAAATAAAACGAAAGGCTCAGTCGAAAGACTGGGCCTTTCGTTTTATCTGTTGTTTGTCG GTGAACGCTCTCTACTAGAGTCACACTGGCTCACCTTCGGGTGGGCCTTTCTGCG		
					*E. coli/Viral*	

pC0.063	T_pSB__1A__K__3_	–11.8	44	ATTTCAGATAAAAAAAATCCTTAGCTTTCGCTAAGGATGATTTC	*E. coli*	Vasudevan et al., 2019

pC0.079	T_psbA__2_	–1.9	83	CCAACTGAATAATCTGCAAATTGCACTCTCCTTCAATGGGGGGTGCTTTTTGCTTGACTG AGTAATCTTCTGATTGCTGATCT		
					PCC 6803	
pC0.081	T_psaB_	–10.6	53	TTAAGCTTGTCCCCTGCCCTCGTTGGTGGGGGATTTGCTTTAATTGGCTGATC		

*The sequences have been annotated with features common to intrinsic terminators, including the A-tract (black underlined), stem (blue), loop (red), and U-tract (green underlined) (see Figure 1C) as reported by Chen et al. (2013). The features for the additional terminators were predicted using ARNold (http://rna.igmors.u-psud.fr/toolbox/arnold), Kinefold (http://kinefold.curie.fr/), or FindTerm (http://www.softberry.com/) (Xayaphoummine et al., 2005; Naville et al., 2011). Gibbs free energy values for the extended hairpin formation (i.e., the A- and U-tract) (ΔG_*A*_) were calculated according to the equation ΔG_*A*_ = ΔG_HA_ − ΔG_*H*_, where ΔG_HA_ is the free energy of the hairpin loop with the inclusion of eight nucleotides upstream and downstream, and ΔG_*H*_ is the free energy of the hairpin loop alone (for generation of ΔG_*HA*_ and ΔG_*H*_ values, see section “Materials and Methods”). Further free energy values are shown in Supplementary Table S4.*

### Validation of the Dual Reporter Testing System in *E. coli*

**FIGURE 2:**
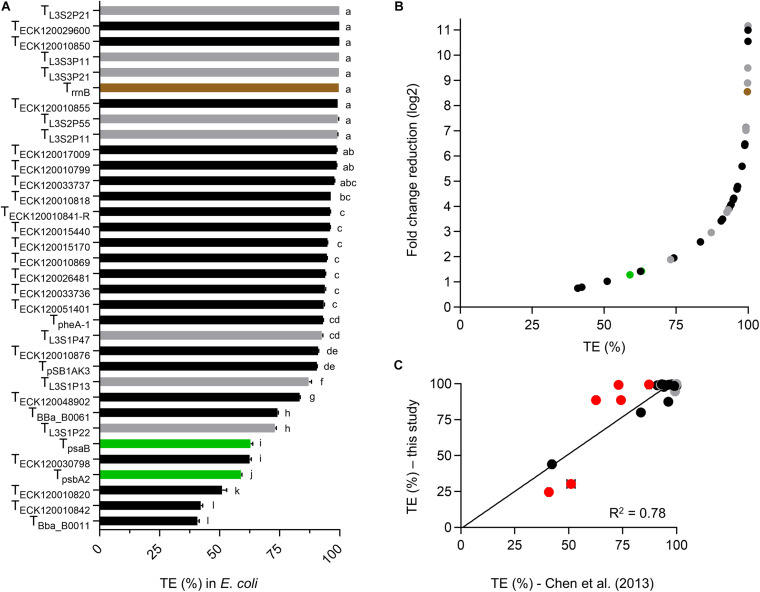
Validation of the dual fluorescent reporter system in *E. coli*. **(A)** TE values for *E. coli* transformants at the early exponential phase of growth. Values are color coded for native *E. coli* (black), synthetic (gray) and native PCC 6803 terminators (green). T_rrnB_ is shown in brown. Error bars represent the ± standard error (SE) of the mean of >10,000 individual cells of four to eight biological replicates. Lowercase letters indicating significant difference (*P* < 0.05) are shown, as determined by ANOVA followed by Tukey’s honestly significant difference test. **(B)** Comparison between TE and corresponding normalized fold change reduction in downstream reporter expression. An increase in one log2 value represents a 2-fold normalized change reduction. **(C)** Correlation analysis of TE values calculated from Chen et al. (2013) (Supplementary Table S3) and TE values determined in this study (*n* = 30). T_rrnB_, T_pSB__1A__K__3_, T_psbA__2_, and T_*psaB*_ were excluded, as data was not available for comparison. The coefficient of determination (R^2^) is shown. Terminator TE values marked in red (T_ECK__12003__0798,_ T_ECK__12001__0820,_ T_Bba_B__0011,_ T_Bba_B__0061,_ T_L__3S1P__22_, and T_L__3S1P__13_) differed from Chen et al. (2013) by more than 10%. Removal of these six terminator from the correlation analysis resulted in *R*^2^ = 0.9).

We first assessed the dual fluorescent reporter system in *E. coli* by generating TE values for each terminator and compared these to the data reported by Chen et al. (2013) (Figure 2A). Terminator strength (TS) values reported by Chen et al. (2013) were converted to a more commonly reported TE (Supplementary Table S3; Hess and Graham, 1990; Yager and von Hippel, 1991; Cambray et al., 2013; Mairhofer et al., 2015).

*E. coli* cultures measured at early exponential growth phase had similar levels of eYFP fluorescence across different strains with an average value of 7034 ± 134 arbitrary units (a.u.) (Supplementary Figure S1). In contrast, the strains showed a wide range of mTagBFP fluorescence values from 1.3 ± 3.4 a.u. to 9094 ± 446 a.u. Both eYFP and mTagBFP fluorescence values showed a unimodal and narrow distribution (Supplementary Figure S2). As expected, the two “no terminator” controls pC1.376 and pC1.377 produced the highest mTagBFP fluorescence values. Previous reports have indicated that translation efficiency is dependent on the length of the transcript (Lim et al., 2011), so we checked if eYFP levels might be decreased in the “no terminator” controls compared to plasmid with terminators. However, we observed no significant differences in eYFP levels between different plasmids, indicating that efficiency of eYFP translation was not reduced in either “no terminator” controls (Supplementary Figure S1B). The mTagBFP:eYFP ratio (i.e., Equation 1) for pC1.376 was 22% higher than for pC1.377, which indicated that pC1.376 produced more transcripts containing both mTagBFP and eYFP. Thus, we decided to use pC1.376 for all TE calculations in this study.

Sixteen terminators had TE values of >95% in *E. coli* (Figure 2A and Supplementary Table S3), with T_L__3S2P__21_ and T_Bba_B__0011_ producing the highest (99.9%) and lowest values (40.8%), respectively. TE values for both PCC 6803 terminators were relatively low in *E. coli* (*ca*. 60%). Overall, the terminator library demonstrated a corresponding 10-fold change reduction in normalized downstream reporter expression (Figure 2B). We then compared the TE values for 30 native *E. coli* and synthetic terminators with those also reported in Chen et al. (2013) and observed a reasonable correlation (coefficient of determination (R^2^) = 0.78), with 19 of the observed TE values differing by less than 5% (Figure 2C). The latter included 14 of the 16 strongest terminators with TE values of >95%. Similarly, the three weakest terminators (T_Bba_B__0011_, T_ECK__120010842_, and T_ECK__120010820_) were the same in both data sets. Six terminators showed a greater difference in TE values (i.e., 12–26%), which comprised four native *E. coli* terminators (T_ECK__12003__0798_, T_ECK__12001__0820_, T_Bba_B__0011_, and T_Bba_B__0061_) and two synthetic terminators (T_L__3S1P__22_ and T_L__3S1P__13_). These variations may have been due to differences in experimental setup (e.g., the vector, origin of replication (ori) and reporter genes) and the different strain of *E. coli* used, as significant differences in the behavior of some terminators has been reported between different *E. coli* strains (Kelly et al., 2019).

### Performance of the Terminator Library in *Synechocystis* sp. PCC 6803

We next evaluated the terminator library in PCC 6803. Due to the slower growth rates of PCC 6803 compared to *E. coli* (Supplementary Figure S3A), we measured fluorescence levels at 24, 48, and 72 h (Supplementary Figure S3B). The cyanobacterial strains grew at comparable rates and the majority expressed eYFP at similar levels between strains at each time point. The single exception was T_L__3S2P__21_, which produced eYFP values consistently 2.5-fold higher than other strains. We are unsure why eYFP values were higher for T_L__3S2P__21_, but we did re-confirm the terminator sequence in this strain by Sanger sequencing. In *E. coli* and bacteriophages, some intrinsic terminators can enhance upstream gene expression by enhancing the stability of the mRNA transcript via the hairpin loop (Abe and Aiba, 1996; Cisneros et al., 1996). Enhancement of mRNA stability by several putative intrinsic terminators has also been demonstrated for the marine species *Synechococcus* sp. PCC 7002, where transcripts with a canonical intrinsic terminator downstream were found to have a longer a half-life compared to transcripts without a downstream terminator (Gordon et al., 2020). However, T_L__3S2P__21_ shares the same U-tract as both T_L__3S2P__11_ and T_L__3S2P__55_ but no increased eYFP expression was observed in the latter strains. mRNA transcript stability is a subject of ongoing research, but some examples of causative factors in heterotrophic bacteria include starvation in *E. coli* and *Lactococcus lactis* (Redon et al., 2005; Morin et al., 2020), and temperature induced stress in *Staphylococcus aureus* and *Mycobacterium tuberculosis* (Anderson et al., 2006; Rustad et al., 2013). mRNA concentration can influence mRNA stability, with increasing transcript concentration leading to decreased stability and mRNA turnover in *E. coli* and *L. lactis* (Nouaille et al., 2017). Similar examples have not been reported yet for PCC 6803.

Similarly to *E. coli*, PCC 6803 strains produced a wide range of mTagBFP fluorescence values at each time point (Supplementary Figure S3B), while the mTagBFP:eYFP ratio for the “no terminator” control pCA1.376 was also consistently higher by 21 ± 2% compared to pCA1.377. A strong correlation was shown between TE values measured at different time points with R^2^ values ranging from 0.982 to 0.988 (Supplementary Figure 3C). Comparison of TE values over the three time points were consistent for strong terminators (Supplementary Table S3). In contrast, weaker terminators tended to show a small decline in TE over time, although there was no significant change in the rankings observed. Overall, terminator behavior in PCC 6803 was consistent between on OD_750_ of 0.4 and 5.9 (Supplementary Table S3). Thus, we focused on reporting TE values at a single time point (48 h) below.

**FIGURE 3:**
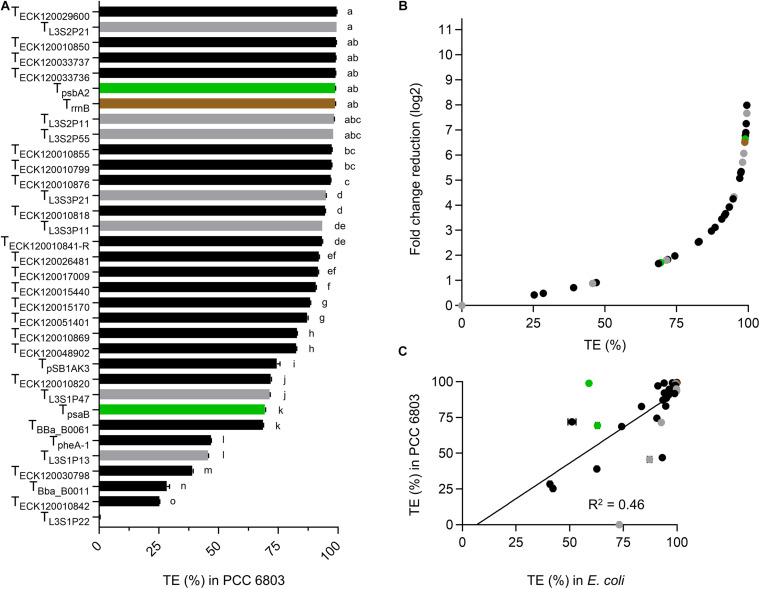
Terminator performances in *Synechocystis* sp. PCC 6803. **(A)** TE values from PCC 6803 transconjugants after 48 h of growth. Color coding is as in Figure 2. Error bars represent the ±SE of the mean of >10,000 individual cells of four biological replicates. Lowercase letters indicating significant difference (*P* < 0.05) are shown, as determined by ANOVA followed by Tukey’s honestly significant difference test. **(B)** Comparison between TE values and corresponding normalized fold change reduction in downstream reporter expression. **(C)** Correlation analysis of TE values between *E. coli* and PCC 6803 (*n* = 34).

Thirteen terminators had TE values of >95% in PCC 6803 (Figure 3A and Supplementary Table S3), with T_L__3S2P__21_ and T_ECK__120029600_ producing the highest value (99.5%) and T_ECK__120010842_ producing the lowest value (25.3%). Ten of the 13 strongest terminators in PCC 6803 also produced TE of >95% in *E. coli* (Figure 2A). Similarly, the two weakest terminators in PCC 6803 (T_ECK__120010842_ and T_Bba_B__0011_) were also the weakest in *E. coli*. Notably, T_L__3S1P__22_ showed no detectable terminator activity in PCC 6803, but had a TE value of 73% in *E. coli*. Overall, the terminator library demonstrated a corresponding 8-fold change reduction in normalized downstream reporter expression in PCC 6803 (Figure 3B). The TE values of 10 terminators differed more widely from those in *E. coli* (i.e., by 12–46%). Thus, the correlation of TE values between *E. coli* and PCC 6803 was modest (*R*^2^ = 0.46) (Figure 3C). Removal of T_L__3S1P__22_ led to only a marginal improvement (*R*^2^ = 0.53).

### Performance of the Terminator Library in *Synechococcus elongatus* UTEX 2973 and Comparison Between Species

Lastly, we evaluated our terminator library in the high-light tolerant strain UTEX 2973. UTEX 2973 generally grew faster than PCC 6803, but showed more variability in growth rates (Supplementary Figure S4A). This was likely due to a greater relative difference in light distribution within the growth incubator under the higher light levels used for culturing UTEX 2973, as strains in the same plate showed more similar rates of growth compared to those located at different positions within the incubator. As for PCC 6803, we measured fluorescence levels for UTEX 2973 at 24, 48, and 72 h (Supplementary Figure S4B). Consistent with the observed differences in growth, the expression levels of eYFP were variable between strains at 24 hr. However, this variation decreased over time.

As for PCC 6803, mTagBFP fluorescence values for the UTEX 2973 strains showed a wide spread at each time point, while the mTagBFP:eYFP ratio for pCA1.376 was consistently higher by 20 ± 5% compared to pCA1.377. Furthermore, the expression levels of mTagBFP and eYFP for pCA1.337 were more variable over time in UTEX 2973, with large increases in both eYFP and mTagBFP fluorescence values observed at 48 h (Supplementary Figure S4B). The TE values over the three time points were similar for most strains, with R^2^ values ranging from 0.964 to 0.978 (Supplementary Figure 4C), indicating that terminator behavior in UTEX 2973 was consistent between an OD_750_ of 0.4–11 (Supplementary Table S3). Thus, as for PCC 6803 we also focused on reporting TE values at 48 h below.

**FIGURE 4:**
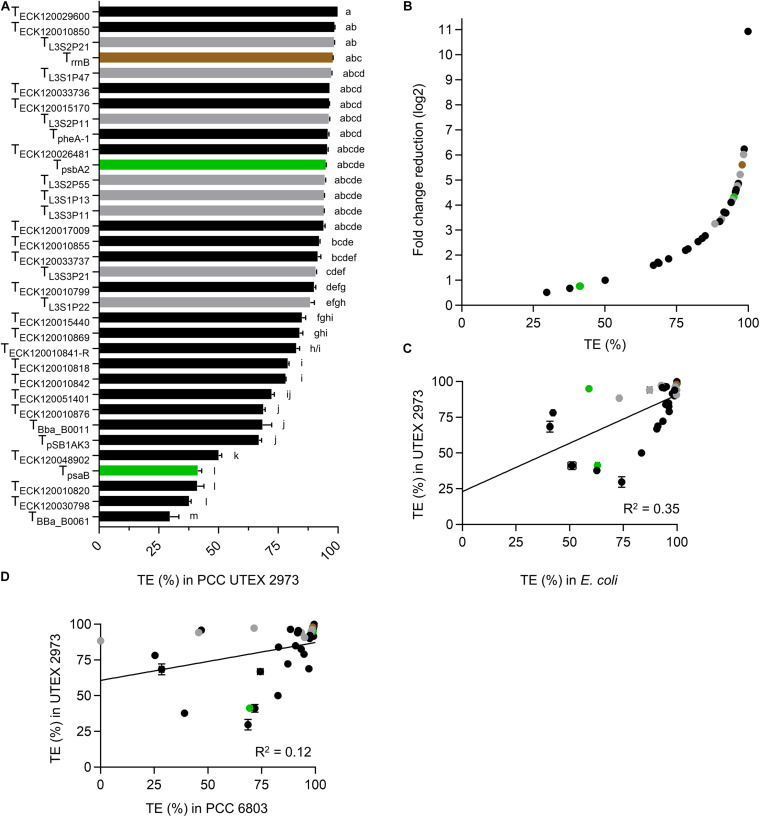
Terminators performances in *Synechococcus elongatus* UTEX 2973. **(A)** TE value from UTEX 2973 after 48 h growth. Color coding is as in Figure 2. Error bars represent the ± SE of the mean of >10,000 individual cells of four biological replicates. Lowercase letters indicating significant difference (*P* < 0.05) are shown, as determined by ANOVA followed by Tukey’s honestly significant difference test. **(B)** Comparison between TE values and corresponding normalized fold change reduction in downstream reporter expression. **(C)** Correlation analysis of TE values between *E. coli* and UTEX 2973 (*n* = 34). **(D)** Correlation analysis of TE values between PCC 6803 and UTEX 2973 (*n* = 34).

Eleven terminators had TE values of >95% in UTEX 2973 (Figure 4A and Supplementary Table S3), with T_ECK__120029600_ producing a very high value of 99.9% and T_Bba_B__0061_ producing the lowest value (29.7%). Six of the 10 strongest terminators in UTEX 2973 produced TE values of >95% in *E. coli* (Figure 2A), while seven of these terminators also produced TE values of >95% in PCC 6803 (Figure 3A). The three weakest terminators in UTEX 2973 (T_Bba_B__0061_, T_ECK__120030798_, and T_ECK__120010820_) were among the bottom ten ranked terminators in PCC 6803 and *E. coli*. T_ECK__120010820_ achieved the same ranking (i.e., 3rd weakest terminator) in both UTEX 2973 and *E. coli*. Overall, the terminator library demonstrated a corresponding 10-fold change reduction of normalized downstream reporter expression in UTEX 2973 (Figure 4B). Similarly to PCC 6803, the correlation of TE values between UTEX 2973 and *E. coli* was low (*R*^2^ = 0.35) (Figure 4C). More surprisingly, the correlation of TE values between UTEX 2973 and PCC 6803 was even lower (*R*^2^ = 0.12) (Figure 4D).

We next compared the TE values for *E. coli*, PCC 6803 and UTEX 2973 to identify terminators that were consistently strong between different species (Supplementary Table S3). The overall strongest terminator was T_ECK__12002__9600_, which had TE values of >99.5% across all three species. A further four terminators (T_L__3S2P__21_, T_ECK__12001__0850_, T_L__3S2P__11_, and T_rrnB_) also had consistent cross-species TE values of >96%. For the two cyanobacterial species alone, T_ECK__120033736_ and T_psbA__2_ had TE values of >95.8%. The TE values for these seven strong terminators was also very consistent over time for PCC 6803 and UTEX 2973.

### The Performance of the Seven Strongest Terminators Was Consistent Under Suboptimal Growth Conditions

To examine if terminator performance might be affected by the growth environment, we measured the TE values for the seven strongest terminators in PCC 6803 and UTEX 2973 grown under suboptimal conditions. Both species were cultured at 30°C in 300 μM photons m^–2^ s^–1^, which is considered high light for PCC 6803 (typically grown at 100 μM photons m^–2^ s^–1^) and a low temperature for UTEX 2973 (typically grown at 40°C) (Vasudevan et al., 2019).

Both PCC 6803 and UTEX 2973 grew at similar rates and reached an OD_750_ of 5.9 and 5.7 after 72 h, respectively (Supplementary Figure S5A). In higher light PCC 6803 grew faster than under typical conditions, while growth rates were reduced in UTEX 2973 due to the lower temperature. Fluorescence measurements for eYFP and mTagBFP in PCC 6803 were comparable to those under typical growth conditions (Supplementary Figure S5B). In contrast, fluorescence values were generally reduced at all time points in UTEX 2973 (Supplementary Figure S5C). TE values for each day were calculated as before (Supplementary Table S3), and the mean values for the three time points were compared (Table 2). Overall, all seven terminators retained TE values of >95.8% for both species under the suboptimal growth conditions, and T_ECK__120029600_ remained the strongest terminator. Overall, our results indicated that the performance of these terminators was generally consistent and robust between the two growth conditions.

**TABLE 2:** Terminator performances in *Synechocystis* sp. PCC 6803 and Synechococcus elongatus UTEX 2973 under suboptimal growth conditions.

	**TE (%) in PCC 6803**		**TE (%) in UTEX 2973**	
	**30°C, 100 μM photons m^–2^ s^–1^**	**30°C, 300 μM photons m^–2^ s^–1^**	**40°C, 300 μM photons m^–2^ s^–1^**	**30°C, 300 μM photons m^–2^ s^–1^**
T_L__3S2P__21_	99.5 ± 0.1	99.6 ± 0.1	97.1 ± 2.2	98.1 ± 0.9
T_L__3S2P__11_	98.3 ± 0.2	98.3 ± 0.6	95.9 ± 0.5	95.9 ± 1.0
T_*ECK120010850*_	99.2 ± 0.2	99.4 ± 0.2	98.1 ± 0.6	98.5 ± 0.3
T_*ECK120033736*_	99.0 ± 0.1	99.3 ± 0.3	97.0 ± 0.9	98.1 ± 1.3
T_*ECK120029600*_	99.6 ± 0.3	99.7 ± 0.2	99.9 ± 0.1	99.9 ± 0.1
T_rrnB_	98.4 ± 0.4	98.4 ± 0.9	98.5 ± 0.5	98.0 ± 0.7
T_*psbA2*_	98.7 ± 0.2	98.8 ± 0.4	95.8 ± 0.9	96.9 ± 1.2

*The mean TE values for PCC 6803 and UTEX 2973 at 24, 48, and 72 h are shown for typical and suboptimal growth conditions (see Supplementary Table S3 for daily values). The standard deviation represents four biological replicates from each time point (n = 12).*

## Discussion

Here, we adapted a dual reporter tool for the CyanoGate MoClo Assembly system that provides a normalized quantification of terminator efficiency within and between species. The pDUOTK1-L1 vector is compatible with several available libraries and thus facilitates easy adoption and sharing of parts with the community (Andreou and Nakayama, 2018; Lai et al., 2018; Valenzuela-Ortega and French, 2019; Vasudevan et al., 2019), and is accessible to any lab currently using Golden Gate cloning. The robustness of our system was validated by comparing results in *E. coli* against previously published data (Chen et al., 2013).

The pDUOTK1-L1 vector contains the broad host range replicative origin RSF1010, which has been shown to be functional in a wide diversity of prokaryotic species, including cyanobacteria from all five subsections (Mermet-Bouvier et al., 1993; Stucken et al., 2012; Bishé et al., 2019). Thus, pDUOTK1-L1 could help to make terminator characterization more accessible, as promising new strains are discovered (Włodarczyk et al., 2019; Jaiswal et al., 2020; Nies et al., 2020). To the best of our knowledge, this is the first study to compare the efficiencies of terminators between two different cyanobacterial species. We identified five strong terminators with consistent TE values in *E. coli*, PCC 6803 and UTEX 2973. These findings should help to inform future strategies for building gene expression systems or more advanced gene circuit designs.

Besides the double terminator T_rrnB_, no unique features could be identified for any of the five strong terminators that behaved consistently between all three species (i.e., the hairpin loop length and GC content, and adenine and uracil content for the A-tract and U-tract, respectively). Overall, our results showed that terminator performances was highly reproducible at different growth points for the same strain but generally differed between the three species examined, and significant differences were observed between PCC 6803 and UTEX 2973 even though both are subsection I species (Castenholz et al., 2001). We also demonstrated that the performance of the seven strongest terminators was consistent in different growth conditions for PCC 6803 and UTEX 2793. Cyanobacterial RNAPs do differ in structure compared to other bacterial RNAPs [for a recent review see Stensjö et al. (2018)]. In addition, RNAP subunits also differ between cyanobacterial species [for a recent review see Srivastava et al. (2020)]. For example, the primary vegetative sigma factor (sigA) in PCC 6803 (srl0653) and UTEX 2973 (WP_071818124.1) have a shared identity and similarity of only 70.5 and 74.1%, respectively (Supplementary Figure S7). Furthermore, cyanobacteria lack transcription elongation factors commonly found in heterotrophic bacteria to restart elongation and for proofreading of transcripts. To compensate, cyanobacterial RNAPs have evolved additional proof-reading and elongation functionalities (Riaz-Bradley et al., 2020). These differences may account for the observed disparity in terminator performance between *E. coli* and cyanobacteria. However, the differences between PCC 6803 and UTEX 2973 were intriguing, and could suggest that RNAP activities differ between cyanobacterial species and/or that other unknown factors are involved.

Several methods and prediction tools exist for the identification and mapping of intrinsic terminators in different species (Carafa et al., 1990; de Hoon et al., 2005; Gardner et al., 2011; Naville et al., 2011; Fritsch et al., 2015; Millman et al., 2017). Traditionally, these approaches have relied on identifying sequence features associated with intrinsic terminators (e.g., the hairpin loop). Previous studies have suggested a relationship between terminator performance and the estimated Gibbs free energy of the extended hairpin (ΔG_*A*_), the U-tract (ΔG_*U*_) and to a lesser extent the hairpin loop (ΔG_*H*_) (Cambray et al., 2013; Chen et al., 2013). In our study, we did not find a strong correlation between TE values and ΔG_*A*,_ ΔG_*H*_ or the estimated Gibbs free energy of the complete terminator sequence (Supplementary Figure S6). Although our terminator library was relatively small, the differences in terminator behavior within and between species indicated that there may be more factors involved in determining intrinsic termination than can be attributed to the properties of individual structural components. For example, the U-tract appears dispensable for intrinsic termination in mycobacteria (Ahmad et al., 2020). Cutting edge approaches utilizing RNA-seq methods have also been applied for the identification of previously unknown terminators in the *E. coli* genome, which go beyond that which has been achieved with previous structural identification models (Ju et al., 2019). In addition, recent work has shown that terminator sequences can be designed as tunable control elements that can be “turned on” to attenuate gene transcription at low temperatures (Roßmanith et al., 2018). With the growing evidence that the structural components of terminators may be malleable depending on species, future work should focus on understanding the combined contributions of terminator components, including those beyond transcriptional control (e.g., modulation of protein expression) for metabolic engineering (Curran et al., 2013; Ito et al., 2020). This may lead to better designs for strong synthetic terminators with consistent cross-species performance. As terminator research and cyanobacterial synthetic biology progresses, tools such as pDUOTK1-L1 will be useful for reliable and convenient determination of terminator efficiency across a broad-host range.

## Data Availability Statement

The original contributions presented in the study are included in the article/Supplementary Material, further inquiries can be directed to the corresponding author/s.

## Author Contributions

GG and AM: conceptualization and writing–original draft preparation. GG: performing the experiments. BW and AM: supervision. All authors: experimental design and writing–review and editing.

## Conflict of Interest

The authors declare that the research was conducted in the absence of any commercial or financial relationships that could be construed as a potential conflict of interest.

## Footnotes

1 www.addgene.org

2 http://parts.igem.org

3 www.addgene.org/kits/mccormick-cyanogate

4 www.addgene.org/51950

5 www.addgene.org/58495

6 http://unafold.rna.albany.edu/?q=mfold
