# Supplementary material for: Evaluation and Comparison of the Efficiency of Transcription Terminators in Different Cyanobacterial Species
Source: Front Microbiol. 2021 Jan 15;11:624011. doi: 10.3389/fmicb.2020.624011 (PMC7843447; doi:10.3389/fmicb.2020.624011)
Supplement: Supplementary file 2 [file Data_Sheet_2.pdf]

Evaluation and comparison of the efficiency of terminators in different cyanobacterial species

Grant A. R. Gale[1,2,3], Baojun Wang[2,3], Alistair J. McCormick[1,2*]

[1] Institute of Molecular Plant Sciences, School of Biological Sciences, University of Edinburgh, Edinburgh EH9 3BF, United Kingdom
[2] Centre for Synthetic and Systems Biology, University of Edinburgh, Edinburgh EH9 3BF, United Kingdom
[3] Institute of Quantitative Biology, Biochemistry and Biotechnology, School of Biological Sciences, University of Edinburgh, Edinburgh EH9 3FF, United Kingdom

Supplementary Material

Supplementary Information S1. Sequence maps (.gb files) of assembled level 0 vectors, ‘no terminator’ control vectors and pDUOTK1-L1. See .zip file.

Supplementary Table S1. List of primers used to generate level 0 terminator vectors and vector assemblies in this study.

Supplementary Table S2. List of level 1 terminator screening vectors used in this study.

Supplementary Table S3. The efficiency of terminators in *E. coli*, and *Synechocystis* sp. PCC 6803 and *Synechococcus* UTEX 2973 at three time points.

Supplementary Table S4. Gibbs free energy values for terminator sequences.

Supplementary Figure S1. Growth and fluorophore expression levels in *E. coli*.

Supplementary Figure S2. Representative distribution of fluorescence expression levels in *E. coli, Synechocystis* sp. PCC 6803 and *Synechococcus* UTEX 2973.

Supplementary Figure S3. Growth and fluorophore expression levels in *Synechocystis* sp. PCC 6803.

Supplementary Figure S4. Growth and fluorophore expression levels in *Synechococcus* UTEX 2973.

Supplementary Figure S5. Growth and fluorophore expression levels in *Synechocystis* sp. PCC 6803 and *Synechococcus* UTEX 2973 under suboptimal growth conditions.

Supplementary Figure S6. Gibbs free energy values for terminator sequences plotted against TE values for *E. coli, Synechocystis* sp. PCC 6803 and *Synechococcus* UTEX 2973.

Supplementary Figure S7. Amino acid alignment of primary vegetative sigma factors from *Synechocystis* sp. PCC 6803 and *Synechococcus* UTEX 2973.

Supplementary Table S1. List of primers used to generate level 0 terminator vectors and vector assemblies in this study. No terminator sequences required domestication (i.e. removal of *BsaI* or *BpiI* sites). All new terminator sequences (1-19) were from Chen et al. (2013). Sequence maps are provided in **Supplemental Information S1**.

Primers used to amplify terminator sequences for assembly into level 0

No.	Vector ID	Terminator	Amplicon length (bp)	Forward primer	Reverse primer
**1**	pC0.291	$T_{L3S2P21}$	93	GTACGAAGACTCGCTTCTCGGTACCAAATTCCAGAAAAGAGGCCTCCCGA	GTACGAAGACTCAGCGGGACCAAAACGAAAAAGGCCCCCCTTTCGGGAGGCCT
**2**	pC0.292	$T_{L3S2P11}$	89	GTACGAAGACTCGCTTCTCGGTACCAAATTCCAGAAAAGAGACGCTT	GTACGAAGACTCAGCGGGACCAAAACGAAAAAAGACGCTCGAAAGCGTCTCTT
**3**	pC0.293	$T_{L3S2P55}$	89	GTACGAAGACTCGCTTCTCGGTACCAAAGACGAACAATAAGACGCTGAAAAG	GTACGAAGACTCAGCGGGACCAAAACGAAAAAAGACGCTTTTCAGCGTCT
**4**	pC0.294	$T_{L3S3P21}$	85	GTACGAAGACTCGCTTCCAATTATTGAAGGCCTCCCTAACGGGGGGCCTT	GTACGAAGACTCAGCGGGGAGACCAGAAACAAAAAAAGGCCCCCCGTT
**5**	pC0.295	$T_{L3S1P13}$	83	GTACGAAGACTCGCTTGACGAACAATAAGGCCTCCCTAACGGGGGGC	GTACGAAGACTCAGCGTTTTGTTATCAATAAAAAAGGCCCCCCGTTAGGG
**6**	pC0.296	$T_{L3S3P11}$	79	GTACGAAGACTCGCTTCCAATTATTGAACACCCTTCGGGGTGTTTT	GTACGAAGACTCAGCGGGGAGACCAGAAACAAAAAAACACCCCGAAG
**7**	pC0.306	$T_{L3S1P22}$	80	GTACGAAGACTCGCTTGACGAACAATAAGGCCGCAAATCGCGGC	GTACGAAGACTCAGCGTTTTGTTATCAATAAAAAAGGCCGCGATTTGCGG
**8**	pC0.307	$T_{L3S1P47}$	84	GTACGAAGACTCGCTTTTTTCGAAAAAAGGCCTCCCAAATCGGG	GTACGAAGACTCAGCGTTTTGTTGCTATAAAAAAAGGCCCCCCGATTTGG
**9**	pC0.308	$T_{ECK120033737}$	89	GTACGAAGACTCGCTTGGAAACACAGAAAAAAGCCCGCACCTGACAGTGC	GTACGAAGACTCAGCGCCTTTGGTCGAAAAAAAAAGCCCGCACTGTCAGGTGC
**10**	pC0.309	$T_{ECK120033736}$	85	GTACGAAGACTCGCTTAACGCATGAGAAAGCCCCCGGAAGATCACCTTCC	GTACGAAGACTCAGCGGCGCAATAAAAAAGCCCCCGGAAGGTGATCTT
**11**	pC0.310	$T_{ECK120010818}$	86	GTACGAAGACTCGCTTGTCAGTTTCACCTGTTTTACGTAAAAACCCGCTTCGGCG	GTACGAAGACTCAGCGCCAAAAGTAAAAACCCGCCGAAGC
**12**	pC0.311	$T_{ECK120015440}$	81	GTACGAAGACTCGCTTTCCGGCAATTAAAAAAGCGGCTAACCACGCCG	GTACGAAGACTCAGCGTGCAGACGTAAAAAAAGCGGCGTGGTTAG
**13**	pC0.312	$T_{ECK120029600}$	122	GTACGAAGACTCGCTTTTCAGCCAAAAAACTTAAGACCGCCGGTCTTGTCCACTACCTTGCAGTAATGCGGTGGACAGGA	GTACGAAGACTCAGCGTTGAGAAGAGAAAAGAAAACCGCCGATCCTGTCCACCGCATTACTGCAAGGTAGTGGACAAGAC
**14**	pC0.313	$T_{ECK120010799}$	92	GTACGAAGACTCGCTTGTTATGAGTCAGGAAAAAAGGCGACAGAGTAATCTGTCG	GTACGAAGACTCAGCGAAAGCAAGCAAAGAAAAAAGGCGACAGATTACTC
**15**	pC0.314	$T_{ECK120010876}$	87	GTACGAAGACTCGCTTTAAGGTTGAAAAATAAAAACGGCGCTAAAAAGCG	GTACGAAGACTCGCTTTAAGGTTGAAAAATAAAAACGGCGCTAAAAAGCG
**16**	pC0.315	$T_{ECK120015170}$	79	GTACGAAGACTCGCTTACAATTTTCGAAAAAACCCGCTTCGGCGG	GTACGAAGACTCAGCGTTTTAGCTATAAAAAAACCCGCCGAAGCGGGT

**17**	pC0.316	$T_{ECK120017009}$	76	GTACGAAGACTCGCTTGATCTAACTAAAAAGGCCGCTCTGCG	GTACGAAGACTCAGCGAGTGAAAAGAAAAAAGGCCGCAGAGCGGCCTTT
**18**	pC0.317	$T_{ECK120051401}$	79	GTACGAAGACTCGCTTCGCAGATAGCAAAAAAGCGCCTTTAGGG	GTACGAAGACTCAGCGCCACCAATGTAAAAAAGCGCCCTAAAGGCGCT
**19**	pC0.318	$T_{ECK120010855}$	74	GTACGAAGACTCGCTTGTAACAACGGAAACCGGCCATTGCGC	GTACGAAGACTCAGCGAGGCCAAAAAAAACCGGCGCAATGGCCG

Additional primers used for vector assembly

	Vector	Primer Name	Amplicon Length (bp)	Primer
**20**	pDUOTK1-L1	pCAT.262-F	995	GATCGAAGACTCACCGACATTTCCCCGAAAAGTGCCAC
**21**	pDUOTK1-L1	pCAT.262-R		GATCGAAGACTCGCTATTACTTGTACAGCTCGTCCATGC
**22**	pDUOTK1-L1	pICH47732_LacZ-F	610	GATCGAAGACTCCCGACTGGAAAGCGGGC
**23**	pDUOTK1-L1	pICH47732_LacZ-R		GATCGAAGACTCCTGCAGCGGCCGCTACAGCGGGAGACCGGATGCCGGGAGCAGACAA
**24**	pDUOTK1-L1	mTagBFP_RBS_Chen-F	871	GATCGAAGACTCGCAGAAAGAGGAGAAATACTAAATGAGCGAACTGATCAAAGAGAACAT
**25**	pDUOTK1-L1	mTagBFP-R		GATCGAAGACTCTCCCCGCAGAAAGGCCCACCC
**26**	pC1.376	pCAT.262-F	979	GATCGAAGACTCACCGACATTTCCCCGAAAAGTGCCAC
**27**	pC1.376	pCAT.262-NTC-R		GATCGAAGACTCGCTATTACTTGTACAGCTCGTCCATGC
**28**	pC1.376	mTagBFP-NTC-F	882	GATCGAAGACTCTAGCGGCCGCTGCAG
**29**	pC1.376	mTagBFP-R		GATCGAAGACTCTCCCCGCAGAAAGGCCCACCC
**30**	pC1.377	rd1.2-F	60	GATCGGTCTCAGCTTCGCCCCCGGAGGCTTTCCCGGG
**31**	pC1.377	rd1.2-R		GATGGGTCTCAAGCGTGATTTGCCCCGGGAAAGCC

Supplementary Table S2. List of level 1 terminator screening vectors assembled for this study. All level 0 terminator parts (see **Table 1**) were assembled into the level 1 acceptor vector pDUOTK1-L1.

Vector ID	Part name
pC1.342	pDUOTK1-L1-T_{pheA-1}
pC1.343	pDUOTK1-L1-$T_{ECK120010850}$
pC1.344	pDUOTK1-L1-$T_{ECK120026481}$
pC1.345	pDUOTK1-L1-$T_{ECK120010842}$
pC1.346	pDUOTK1-L1-$T_{ECK120048902}$
pC1.347	pDUOTK1-L1-T_{rrnB}
pC1.348	pDUOTK1-L1-$T_{L3S2P21}$
pC1.349	pDUOTK1-L1-$T_{L3S2P11}$
pC1.350	pDUOTK1-L1-$T_{L3S2P55}$
pC1.351	pDUOTK1-L1-$T_{L3S3P21}$
pC1.352	pDUOTK1-L1-$T_{L3S1P13}$
pC1.353	pDUOTK1-L1-$T_{L3S3P11}$
pC1.354	pDUOTK1-L1-$T_{L3S1P22}$
pC1.355	pDUOTK1-L1-$T_{L3S1P47}$
pC1.356	pDUOTK1-L1-$T_{ECK120033737}$
pC1.357	pDUOTK1-L1-$T_{ECK120033736}$
pC1.358	pDUOTK1-L1-$T_{ECK120010818}$
pC1.359	pDUOTK1-L1-$T_{ECK120015440}$
pC1.360	pDUOTK1-L1-$T_{ECK120029600}$
pC1.361	pDUOTK1-L1-$T_{ECK120010799}$
pC1.362	pDUOTK1-L1-$T_{ECK120010876}$
pC1.363	pDUOTK1-L1-$T_{ECK120015170}$
pC1.364	pDUOTK1-L1-$T_{ECK120017009}$
pC1.365	pDUOTK1-L1-$T_{ECK120051401}$
pC1.366	pDUOTK1-L1-$T_{ECK120010855}$
pC1.367	pDUOTK1-L1-T_{Bba_B0011}
pC1.368	pDUOTK1-L1- $T_{pSB1AK3}$
pC1.369	pDUOTK1-L1-$T_{ECK120010820}$
pC1.370	pDUOTK1-L1-T_{BBa_B0061}
pC1.371	pDUOTK1-L1-$T_{ECK120030798}$
pC1.372	pDUOTK1-L1-$T_{ECK120010869}$
pC1.373	pDUOTK1-L1-$T_{ECK120010841-R}$
pC1.374	pDUOTK1-L1-T_{psbA2}
pC1.375	pDUOTK1-L1-T_{psaB}
pC1.376	pDUOTK1-L1-No Term (Chen)
pC1.377	pDUOTK1-L1-No Term (Cambray spacer rd1.2)

Supplementary Table S3. The efficiency of terminators in *E. coli*, and *Synechocystis* sp. PCC 6803 and *Synechococcus* UTEX 2973 at three time points. To compare our measured TE values with data in Chen et al. (2013), we converted the reported terminator strength (TS) values to TE values to allow for meaningful comparison. TS is a non-linear derivative of TE, such that TS values tend to infinity as TE values approaches 100%. Thus, small increases when TE values are >95% result in large increases in TS values. We used the formula $TE = 100 \text{ x } 1 - \frac{1}{\text{TS}}$ to convert TS values to TE values, where TE is termination efficiency (%) and TS is termination strength (a.u.). Both TS values and calculated TE values are shown for Chen et al., (2013) below. TE values are shown for *E. coli*, PCC 6803 and UTEX 2973 measured under typical growth conditions (i.e. 37°C for *E. coli*, 30°C and 100 μM photons m^{-2} s^{-1} for PCC 6803, 40°C and 300 μM photons m^{-2} s^{-1} for UTEX 2973). A correlation matrix table is shown for PCC 6803 and UTEX 2973 at different time points under typical growth conditions. TE values are also shown for the suboptimal growth condition experiment (30°C and 300 μM photons m^{-2} s^{-1} for PCC 6803 and UTEX 2973) (**Supplementary Figure S5**).

**Typical growth conditions**		***E. coli* Chen et al., 2013**		***E. coli* this study**	**PCC 6803**			**UTEX 2973**		
**Vector ID**	**Terminator**	**TS**	**TE (%)**	**TE (%)**	**24 hours TE (%)**	**48 hours TE (%)**	**72 hours TE (%)**	**24 hours TE (%)**	**48 hours TE (%)**	**72 hours TE (%)**
pC0.291	$T_{L3S2P21}$	382.1	99.7	99.9	99.6	99.5	99.5	97.8	98.5	95.1
pC0.292	$T_{L3S2P11}$	261.5	99.6	99.2	98.1	98.5	98.2	95.9	96.2	95.6
pC0.293	$T_{L3S2P55}$	18.6	94.6	99.3	97.5	98.1	98.3	83.1	94.5	90.1
pC0.294	$T_{L3S3P21}$	246.6	99.6	99.8	95.7	95.0	95.1	83.5	90.7	87.4
pC0.295	$T_{L3S1P13}$	177.9	99.4	87.2	51.3	45.7	40.7	96.4	94.2	95.9
pC0.296	$T_{L3S3P11}$	172.6	99.4	99.9	93.6	93.5	92.6	97.3	94.2	97.6
pC0.306	$T_{L3S1P22}$	128.1	99.2	73.1	4.4	0.0	0.0	89.8	88.4	93.0
pC0.307	$T_{L3S1P47}$	123.4	99.2	92.7	74.2	71.5	68.2	95.4	97.2	96.7
pC0.066	$T_{pheA\text{-}1}$	243.5	99.6	93.1	49.8	47.0	40.2	96.5	95.8	96.7
pC0.068	$T_{ECK120010850}$	64.7	98.5	99.9	99.3	99.3	99.1	97.9	98.7	98.0
pC0.069	$T_{ECK120026481}$	45.9	97.8	94.1	92.8	92.1	87.1	93.9	95.6	93.9
pC0.072	$T_{ECK120010842}$	1.8	44.1	42.2	27.3	25.3	26.1	80.3	78.1	78.9
pC0.074	$T_{ECK120048902}$	5.0	80.0	83.4	84.2	82.6	81.1	55.6	50.1	53.0
pC0.062	T_{Bba_B0011}	1.3	24.7	40.8	34.0	28.5	19.7	69.7	68.5	66.9
pC0.064	$T_{ECK120010820}$	1.4	30.3	51.1	72.8	71.9	70.4	39.8	41.1	39.8

pC0.070	T_{BBa_B0061}	8.8	88.7	74.2	72.6	68.7	64.8	26.3	29.7	25.2
pC0.071	$T_{ECK120030798}$	8.8	88.7	62.6	43.7	39.1	43.5	39.7	37.7	28.5
pC0.073	$T_{ECK120010869}$	83.7	98.8	94.8	85.1	82.9	76.8	86.4	84.0	85.5
pC0.077	$T_{ECK120010841-R}$	8.0	87.5	96.2	93.7	93.4	91.8	85.3	82.6	85.1
pC0.308	$T_{ECK120033737}$	312.5	99.7	97.9	98.7	99.2	98.9	94.5	74.1	95.0
pC0.309	$T_{ECK120033736}$	164.6	99.4	94.0	98.8	99.1	99.1	98.2	96.6	96.2
pC0.310	$T_{ECK120010818}$	148.3	99.3	96.4	95.3	94.8	95.4	80.9	79.0	80.8
pC0.311	$T_{ECK120015440}$	119.2	99.2	96.1	92.2	90.8	90.5	82.7	85.0	82.4
pC0.312	$T_{ECK120029600}$	378.4	99.7	99.9	99.2	99.5	99.9	99.7	99.9	99.9
pC0.313	$T_{ECK120010799}$	101.0	99.0	98.8	97.8	97.4	97.1	93.3	90.1	93.6
pC0.314	$T_{ECK120010876}$	97.4	99.0	91.1	96.8	97.0	97.4	75.3	68.8	65.5
pC0.315	$T_{ECK120015170}$	85.8	98.8	95.0	90.8	88.5	85.5	98.1	96.4	95.7
pC0.316	$T_{ECK120017009}$	67.6	98.5	98.9	92.6	91.7	89.3	91.2	94.0	87.7
pC0.317	$T_{ECK120051401}$	67.2	98.5	93.4	88.7	87.2	83.1	68.4	72.2	64.9
pC0.318	$T_{ECK120010855}$	65.4	98.5	99.3	97.7	97.5	97.3	96.1	92.2	95.1
pC0.082	T_{rrnB}	N/A	N/A	99.7	97.9	98.8	98.4	99.0	97.9	98.6
pC0.063	$T_{pSB1AK3}$	N/A	N/A	90.7	78.7	74.4	65.8	62.3	66.9	50.9
pC0.079	T_{psbA2}	N/A	N/A	59.0	98.5	99.0	98.5	96.7	95.0	95.6
pC0.081	T_{psaB}	N/A	N/A	62.9	69.9	69.5	62.9	49.7	41.4	44.3

Correlation matrix table for PCC 6803

hours	24	48	72
24	1		
48	0.999	1	
72	0.991	0.994	1

Correlation matrix table for UTEX 2973

hours	24	48	72
24	1		
48	0.982	1	
72	0.988	0.982	1

**Suboptimal growth conditions**		**PCC 6803**			**UTEX 2973**		
**Vector ID**	**Terminator**	**24 hours TE (%)**	**48 hours TE (%)**	**72 hours TE (%)**	**24 hours TE (%)**	**48 hours TE (%)**	**72 hours TE (%)**
pC0.291	$T_{L3S2P21}$	99.5	99.7	99.7	99.2	97.4	97.6
pC0.292	$T_{L3S2P11}$	97.5	98.9	98.3	94.8	96.2	96.8
pC0.068	$T_{ECK120010850}$	99.2	99.5	99.5	98.6	98.3	98.5
pC0.309	$T_{ECK120033736}$	99.0	99.5	99.5	99.4	96.4	98.6
pC0.312	$T_{ECK120029600}$	99.5	99.8	99.9	99.9	99.9	99.9
pC0.082	T_{rrnB}	97.2	99.1	98.8	98.9	97.7	97.3
pC0.079	T_{psbA2}	98.3	98.8	99.3	98.0	95.5	97.2

Supplementary Table S4. Gibbs free energy values for terminator sequences. Free energy values are shown for the hairpin loop with the inclusion of the 8 nucleotides directly upstream and downstream (ΔG_{HA}), the hairpin loop alone (ΔG_{H}) and the complete terminator sequence (ΔG_{TERM}). Free energy values were calculated using mFold (http://unafold.rna.albany.edu/?q=mfold) and are given in kcal/mol (Zuker, 2003).

Terminator	ΔG_{HA}	ΔG_{H}	ΔG_{TERM}
$T_{L3S2P21}$	-30.8	-19.8	-37.9
$T_{L3S2P11}$	-18.4	-7.4	-25.5
$T_{L3S2P55}$	-12.6	-9.8	-25.8
$T_{L3S3P21}$	-22.1	-18.0	-23.3
$T_{L3S1P13}$	-20.8	-18.0	-22.5
$T_{L3S3P11}$	-14.3	-10.1	-15.5
$T_{L3S1P22}$	-14.5	-11.7	-16.2
$T_{L3S1P47}$	-24.5	-16.1	-24.5
$T_{pheA\text{-}1}$	-18.9	-16.1	-20.6
$T_{ECK120010850}$	-15.2	-10.8	-15.2
$T_{ECK120026481}$	-18.5	-12.2	-18.5
$T_{ECK120010842}$	-3.6	-1.1	-7.2
$T_{ECK120048902}$	-16.2	-8.3	-17.5
T_{Bba_B0011}	-10.0	-4.5	-11.2
$T_{ECK120010820}$	-11.4	-6.1	-11.4
T_{BBa_B0061}	-24.0	-10.9	-25.0
$T_{ECK120030798}$	-18.2	-12.3	-18.2
$T_{ECK120010869}$	-17.2	-11.6	-17.3
$T_{ECK120010841\text{-}R}$	-9.6	-6.6	-9.6
$T_{ECK120033737}$	-23.7	-15.7	-25.0
$T_{ECK120033736}$	-32.3	-23.6	-37.8
$T_{ECK120010818}$	-22.4	-11.6	-22.4
$T_{ECK120015440}$	-14.4	-8.0	-17.4
$T_{ECK120029600}$	-42.0	-37.2	-42.0
$T_{ECK120010799}$	-27.9	-17.3	-28.9
$T_{ECK120010876}$	-17.5	-11.9	-17.6
$T_{ECK120015170}$	-20.1	-11.6	-20.1
$T_{ECK120017009}$	-15.6	-10.1	-16.2
$T_{ECK120051401}$	-15.8	-8.4	-15.8
$T_{ECK120010855}$	-13.6	-7.9	-17.3
T_{rrnB}	-30.9	-18.7	-60.7
$T_{pSB1AK3}$	-23.2	-11.4	-30.5
T_{psbA2}	-12.9	-11.0	-22.7
T_{psaB}	-21.6	-11.0	-22.7

A

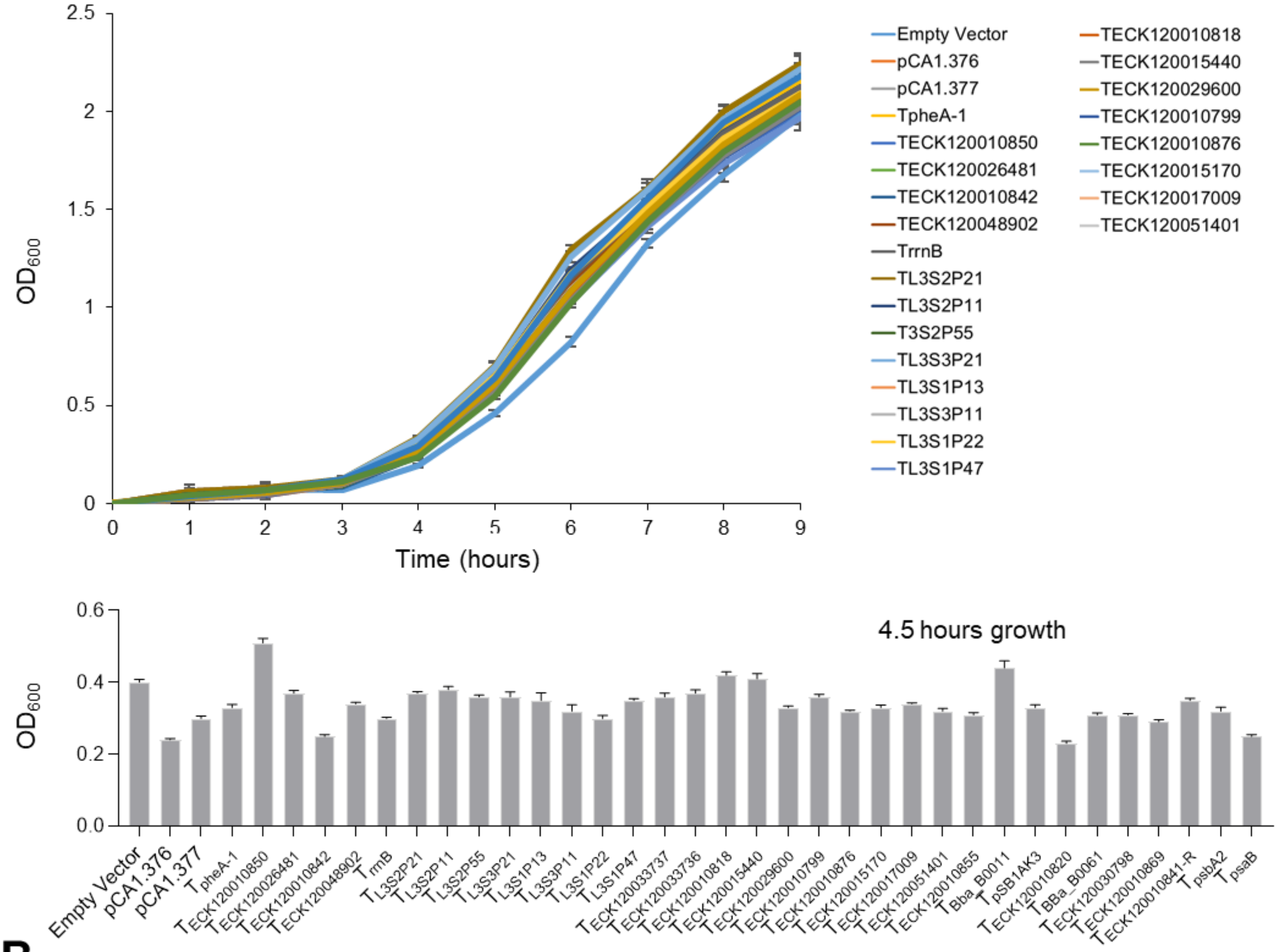
2.5
2
1.5
1
0.5
0
OD600
0
1
2
3
4
5
6
7
8
9
Time (hours)
Empty Vector
pCA1.376
pCA1.377
TpheA-1
TECK120010850
TECK120026481
TECK120010842
TECK120048902
TrrnB
TL3S2P21
TL3S2P11
T3S2P55
TL3S3P21
TL3S1P13
TL3S3P11
TL3S1P22
TL3S1P47
TECK120010818
TECK120015440
TECK120029600
TECK120010799
TECK120010876
TECK120015170
TECK120017009
TECK120051401
0.6
0.4
0.2
0.0
OD600
4.5 hours growth
Empty Vector
pCA1.376
pCA1.377
TpheA-1
TECK120010850
TECK120026481
TECK120010842
TECK120048902
TrrnB
TL3S2P21
TL3S2P11
TL3S2P55
TL3S3P21
TL3S1P13
TL3S3P11
TL3S1P22
TL3S1P47
TECK120033737
TECK120033736
TECK120010818
TECK120015440
TECK120029600
TECK120010799
TECK120010876
TECK120015170
TECK120017009
TECK120051401
TECK120010855
TBba_B0011
TpSB1AK3
TECK120010820
TBBa_B0061
TECK120030798
TECK120010869
TECK120010841-R
TpsbA2
TpsaB

B

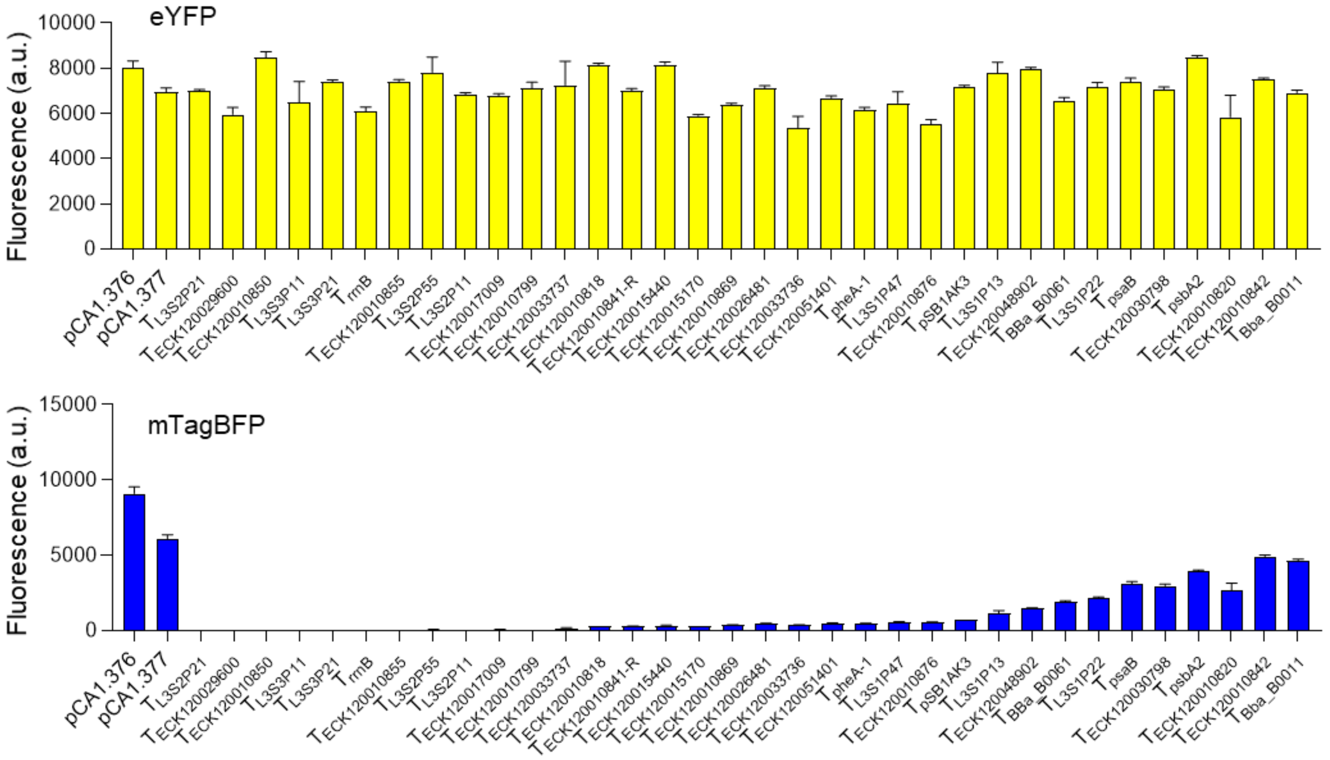
eYFP
10000
8000
6000
4000
2000
0
Fluorescence (a.u.)
pCA1.376
pCA1.377
TL3S2P21
TECK120029600
TECK120010850
TL3S3P11
TL3S3P21
TrrnB
TECK120010855
TL3S2P55
TL3S2P11
TECK120017009
TECK120010799
TECK120033737
TECK120010818
TECK120010841-R
TECK120015440
TECK120015170
TECK120010869
TECK120026481
TECK120033736
TECK120051401
TpheA-1
TL3S1P47
TECK120010876
TpSB1AK3
TL3S1P13
TECK120048902
TBBa_B0061
TL3S1P22
TpsaB
TECK120030798
TpsbA2
TECK120010820
TECK120010842
TBba_B0011
mTagBFP
15000
10000
5000
0
Fluorescence (a.u.)
pCA1.376
pCA1.377
TL3S2P21
TECK120029600
TECK120010850
TL3S3P11
TL3S3P21
TrrnB
TECK120010855
TL3S2P55
TL3S2P11
TECK120017009
TECK120010799
TECK120033737
TECK120010818
TECK120010841-R
TECK120015440
TECK120015170
TECK120010869
TECK120026481
TECK120033736
TECK120051401
TpheA-1
TL3S1P47
TECK120010876
TpSB1AK3
TL3S1P13
TECK120048902
TBBa_B0061
TL3S1P22
TpsaB
TECK120030798
TpsbA2
TECK120010820
TECK120010842
TBba_B0011

Supplementary Figure S1. Growth and fluorophore expression levels in *E. coli*. **(A)** Growth experiment conducted with a reduced number of transformants to identify the beginning of the exponential phase. Values are the means ± standard error (SE) of the mean from 4 biological replicates. OD_{600} values are shown at 4.5 hours growth for the full growth experiment. Average OD_{600} = 0.33±0.01 (n = 37). **(B)** Expression levels of eYFP and mBFP after 4.5 hours growth. Error bars represent the ±SE of the mean of 4-8 biological replicates, where each replicate represents the median measurement of at least 10,000 cells measured by flow cytometry.

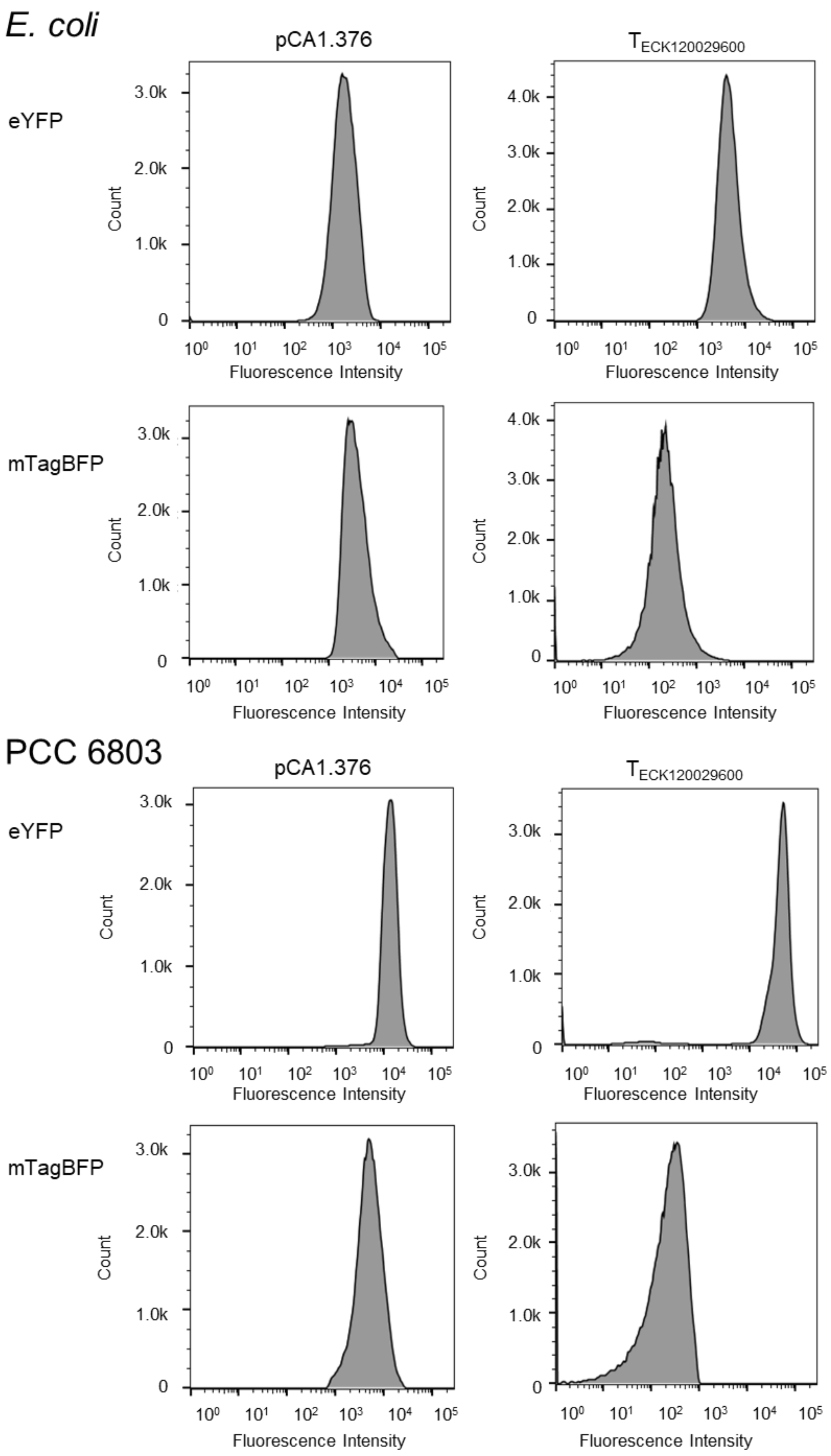
E. coli
pCA1.376
$T_{ECK120029600}$
eYFP
mTagBFP
Count
Fluorescence Intensity
PCC 6803
pCA1.376
$T_{ECK120029600}$
eYFP
mTagBFP
Count
Fluorescence Intensity

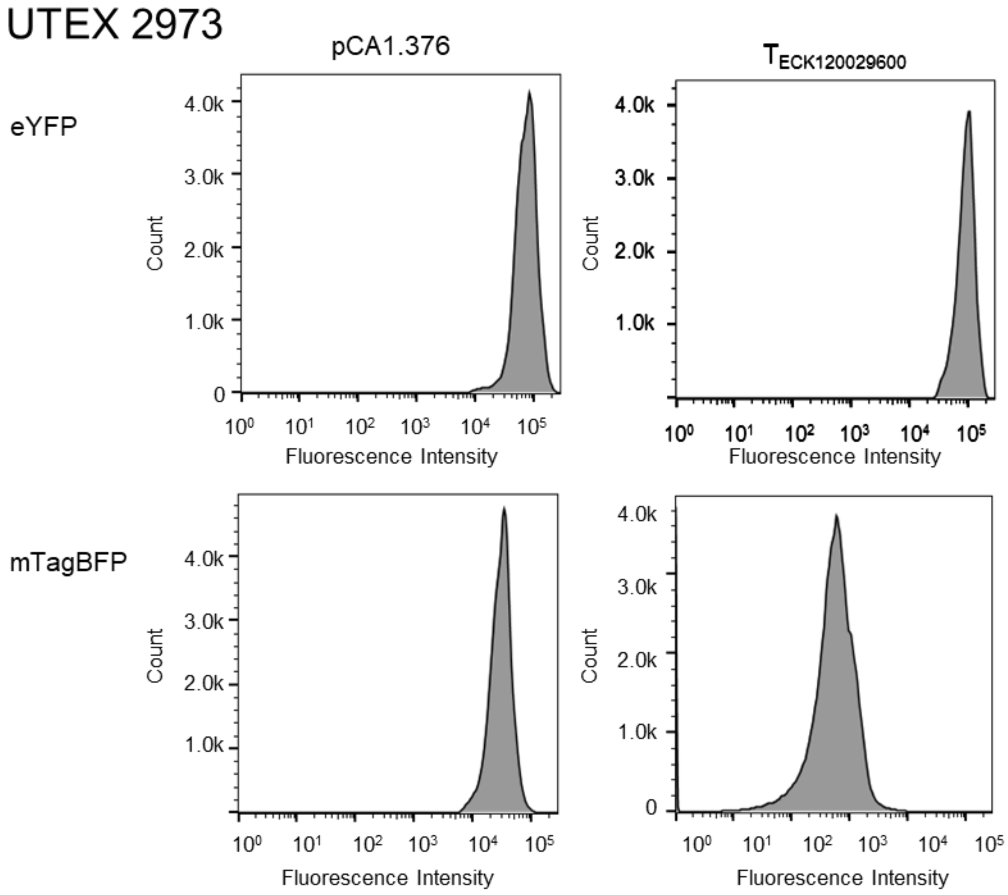

Supplementary Figure S2. Representative distribution of fluorescence expression levels in *E. coli*, *Synechocystis* sp. PCC 6803 and *Synechococcus* UTEX 2973. Populations of at least 10,000 cells were first gated by forward and side scatter (see Materials and Methods). Then histogram plots were generated, and the median fluorescence values extracted.

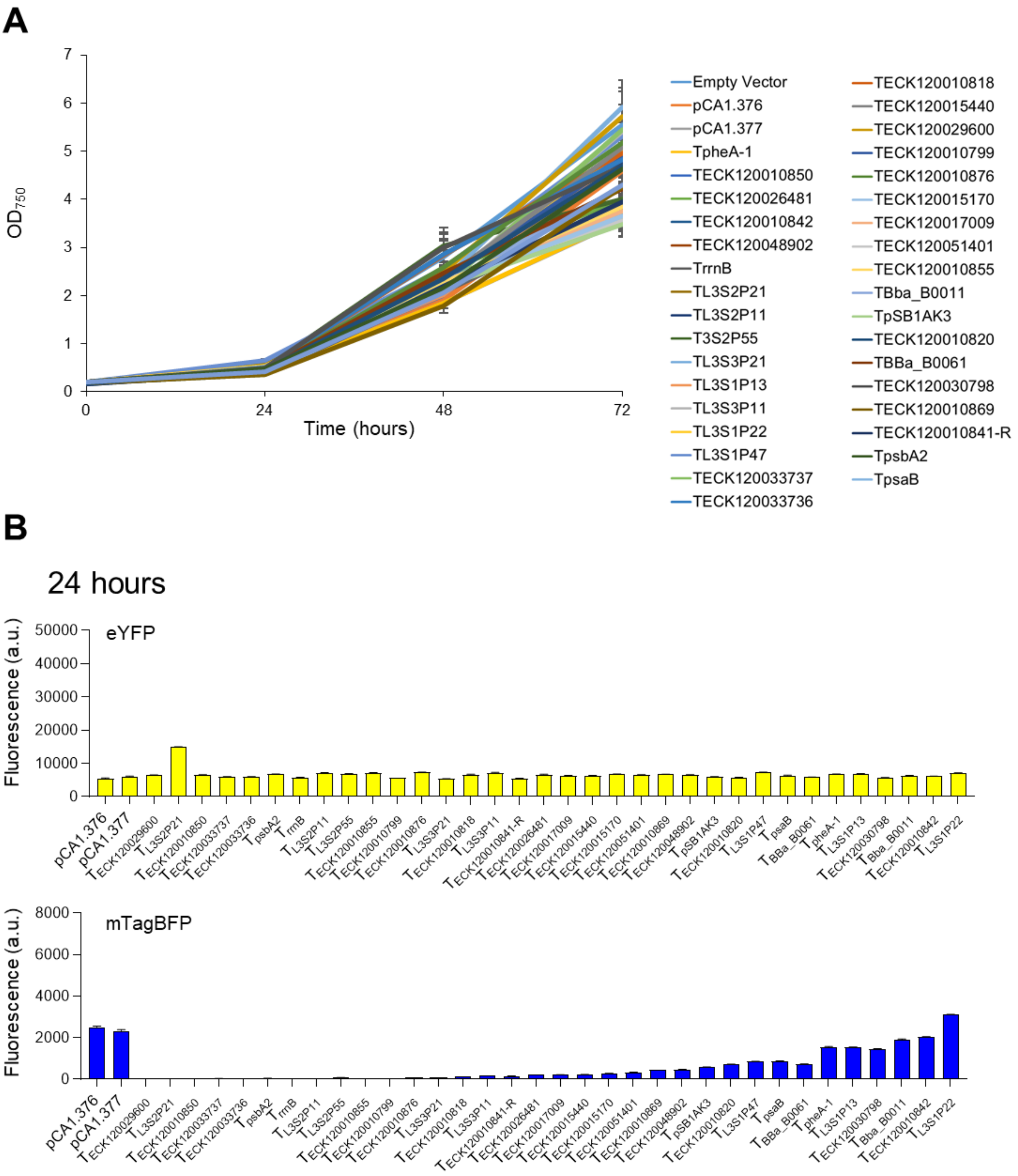
A
OD750
Time (hours)
0
24
48
72
7
6
5
4
3
2
1
0
Empty Vector
pCA1.376
pCA1.377
TpheA-1
TECK120010850
TECK120026481
TECK120010842
TECK120048902
TrrnB
TL3S2P21
TL3S2P11
T3S2P55
TL3S3P21
TL3S1P13
TL3S3P11
TL3S1P22
TL3S1P47
TECK120033737
TECK120033736
TECK120010818
TECK120015440
TECK120029600
TECK120010799
TECK120010876
TECK120015170
TECK120017009
TECK120051401
TECK120010855
TBba_B0011
TpSB1AK3
TECK120010820
TBBa_B0061
TECK120030798
TECK120010869
TECK120010841-R
TpsbA2
TpsaB
B
24 hours
eYFP
Fluorescence (a.u.)
50000
40000
30000
20000
10000
0
mTagBFP
Fluorescence (a.u.)
8000
6000
4000
2000
0
pCA1.376
pCA1.377
TECK120029600
TL3S2P21
TECK120010850
TECK120033737
TECK120033736
TpsbA2
TrrnB
TL3S2P11
TL3S2P55
TECK120010855
TECK120010799
TECK120010876
TL3S3P21
TECK120010818
TL3S3P11
TECK120010841-R
TECK120026481
TECK120017009
TECK120015440
TECK120015170
TECK120051401
TECK120010869
TECK120048902
TpSB1AK3
TECK120010820
TL3S1P47
TpsaB
TBBa_B0061
TpheA-1
TL3S1P13
TECK120030798
TBba_B0011
TECK120010842
TL3S1P22

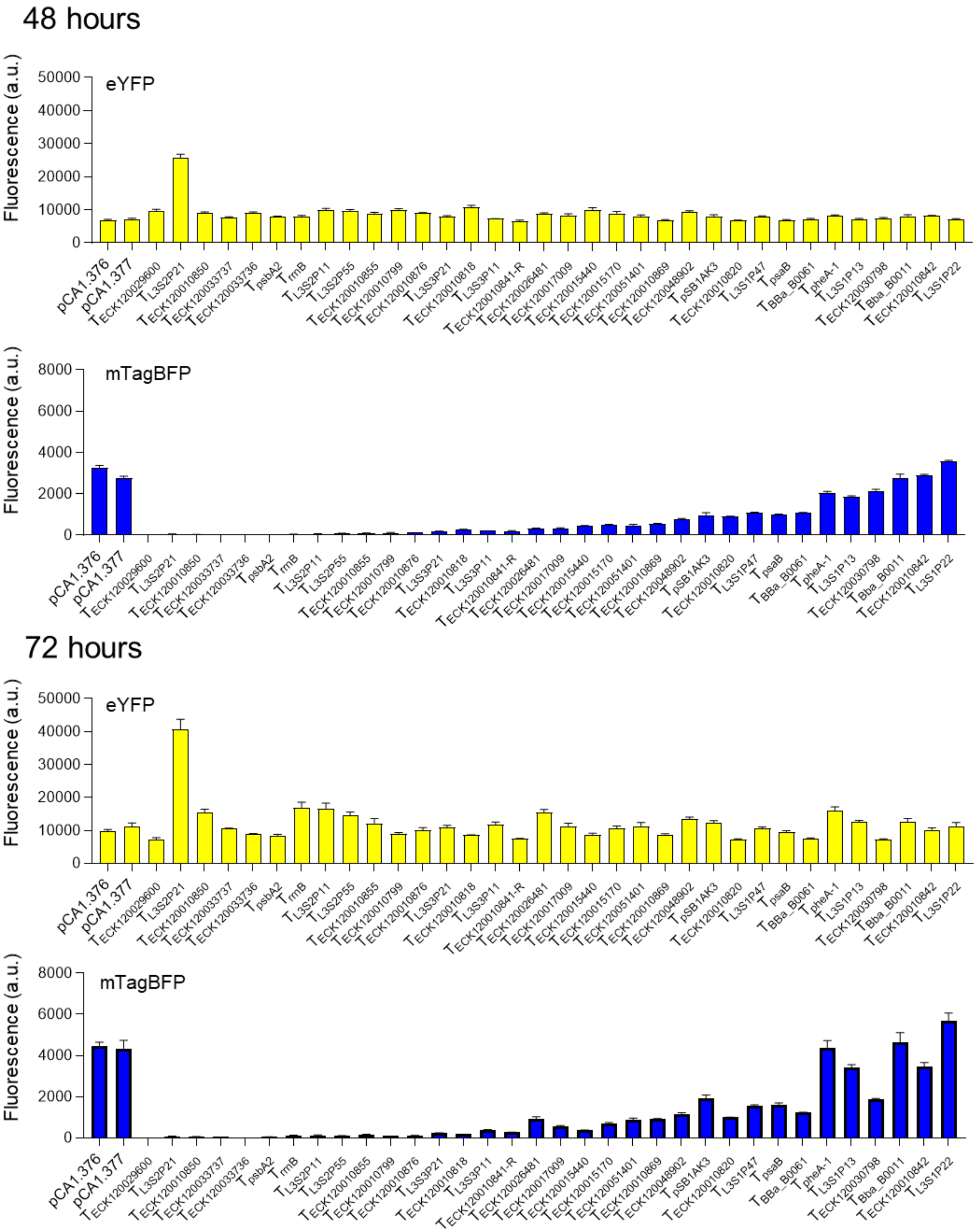
48 hours
eYFP
Fluorescence (a.u.)
50000
40000
30000
20000
10000
0
mTagBFP
8000
6000
4000
2000
0
72 hours
pCA1.376
pCA1.377
TECK120029600
TL3S2P21
TECK120010850
TECK120033737
TECK120033736
TpsbA2
TrrnB
TL3S2P11
TL3S2P55
TECK120010855
TECK120010799
TECK120010876
TL3S3P21
TECK120010818
TL3S3P11
TECK120010841-R
TECK120026481
TECK120017009
TECK120015440
TECK120015170
TECK120051401
TECK120010869
TECK120048902
TpSB1AK3
TECK120010820
TL3S1P47
TpsaB
TBBa_B0061
TpheA-1
TL3S1P13
TECK120030798
TBba_B0011
TECK120010842
TL3S1P22

C

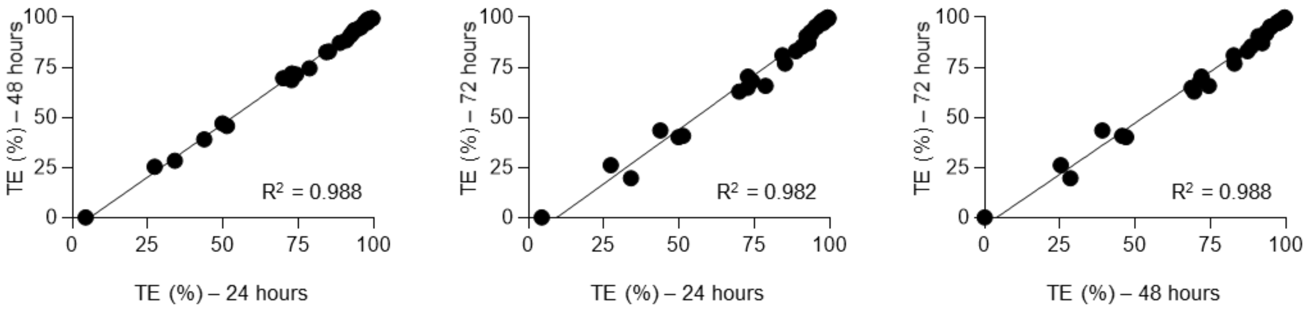

Supplementary Figure S3. Growth and fluorophore expression levels in *Synechocystis* sp. PCC 6803. **(A)** Cultures were grown for 72 hr at 30°C with continuous light (100 µmol photons m^{-2} s^{-1}). **(B)** Expression levels of eYFP and mTagBFP at three time points (24, 48 and 72 hr). Error bars represent the ±SE of the mean of four biological replicates, where each replicate represents the median measurement of at least 10,000 cells measured by flow cytometry. **(C)** Correlation analysis of TE values between the three different time points (n = 34). The coefficient of determination (R^2) is shown.

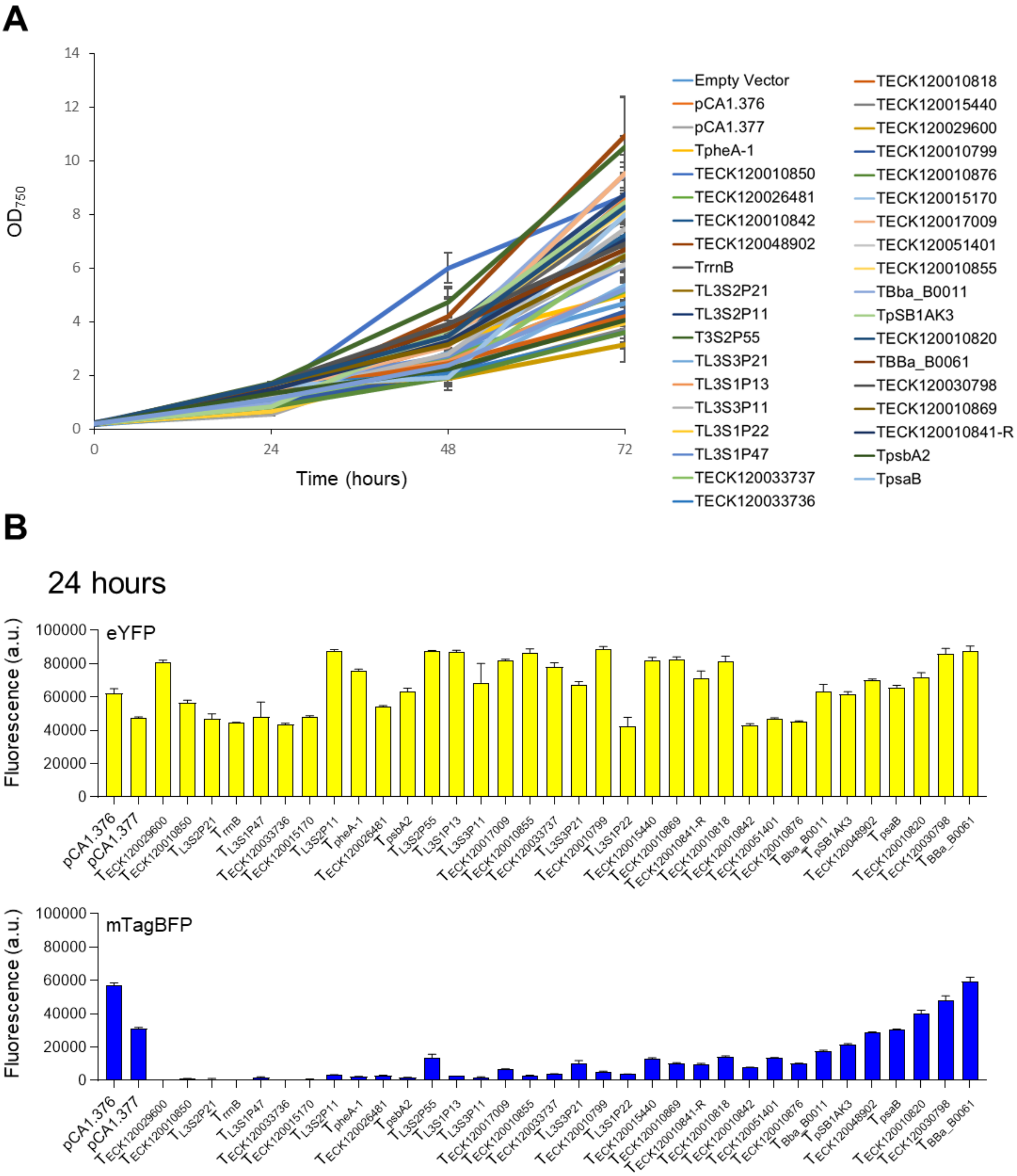
A
OD750
Time (hours)
Empty Vector
pCA1.376
pCA1.377
TpheA-1
TECK120010850
TECK120026481
TECK120010842
TECK120048902
TrrnB
TL3S2P21
TL3S2P11
T3S2P55
TL3S3P21
TL3S1P13
TL3S3P11
TL3S1P22
TL3S1P47
TECK120033737
TECK120033736
TECK120010818
TECK120015440
TECK120029600
TECK120010799
TECK120010876
TECK120015170
TECK120017009
TECK120051401
TECK120010855
TBba_B0011
TpSB1AK3
TECK120010820
TBBa_B0061
TECK120030798
TECK120010869
TECK120010841-R
TpsbA2
TpsaB
B
24 hours
eYFP
mTagBFP
Fluorescence (a.u.)
pCA1.376
pCA1.377
TECK120029600
TECK120010850
TL3S2P21
TrrnB
TL3S1P47
TECK120033736
TECK120015170
TL3S2P11
TpheA-1
TECK120026481
TpsbA2
TL3S2P55
TL3S1P13
TL3S3P11
TECK120017009
TECK120010855
TECK120033737
TL3S3P21
TECK120010799
TL3S1P22
TECK120015440
TECK120010869
TECK120010841-R
TECK120010818
TECK120010842
TECK120051401
TECK120010876
TBba_B0011
TpSB1AK3
TECK120048902
TpsaB
TECK120010820
TECK120030798
TBBa_B0061

48 hours

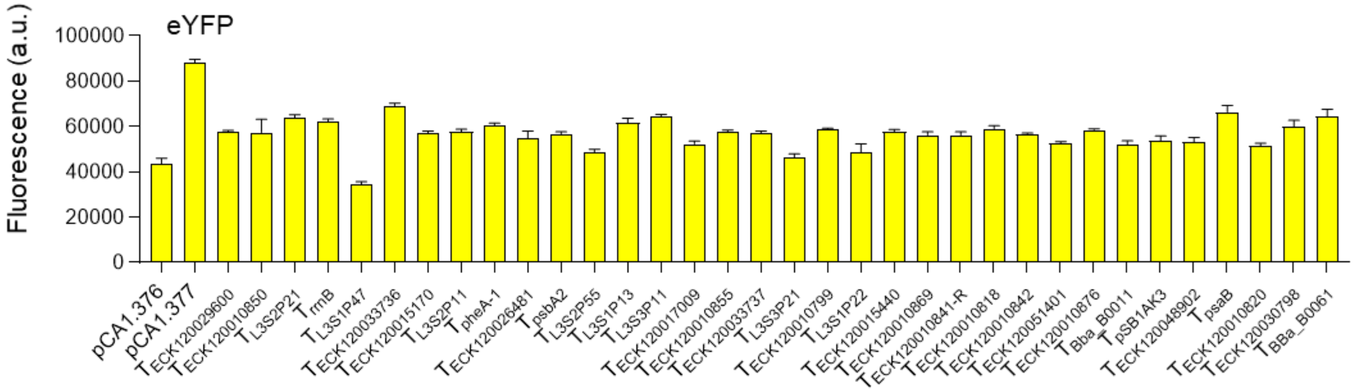

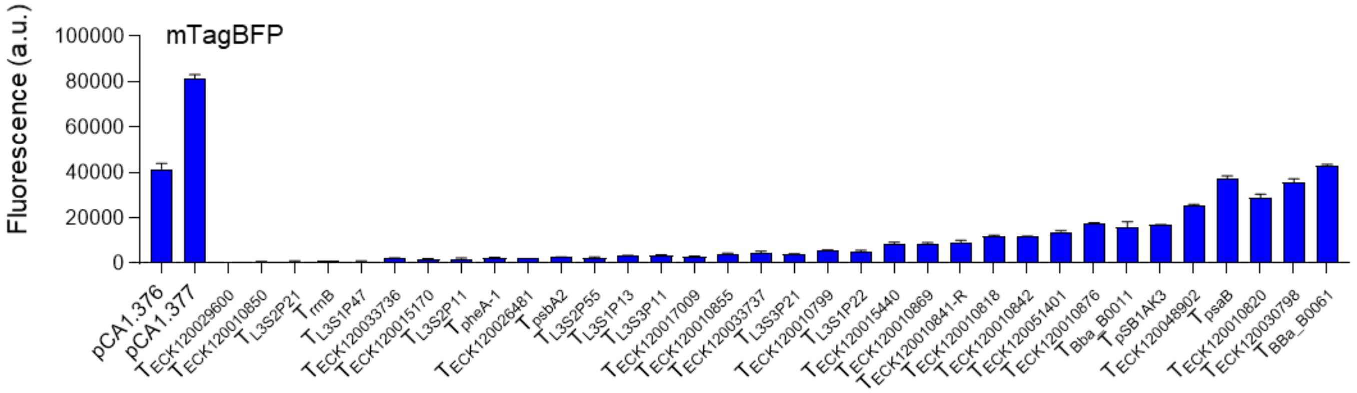

72 hours

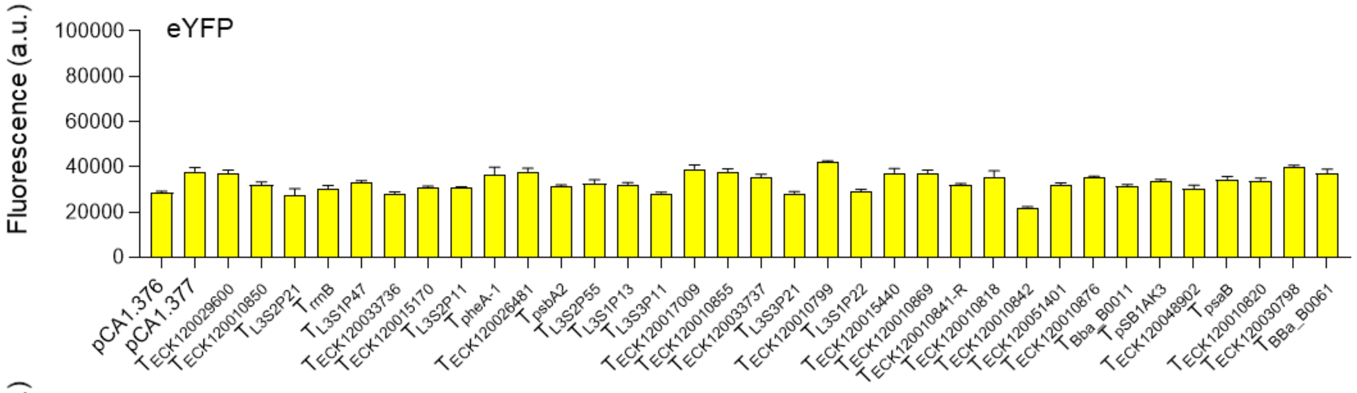

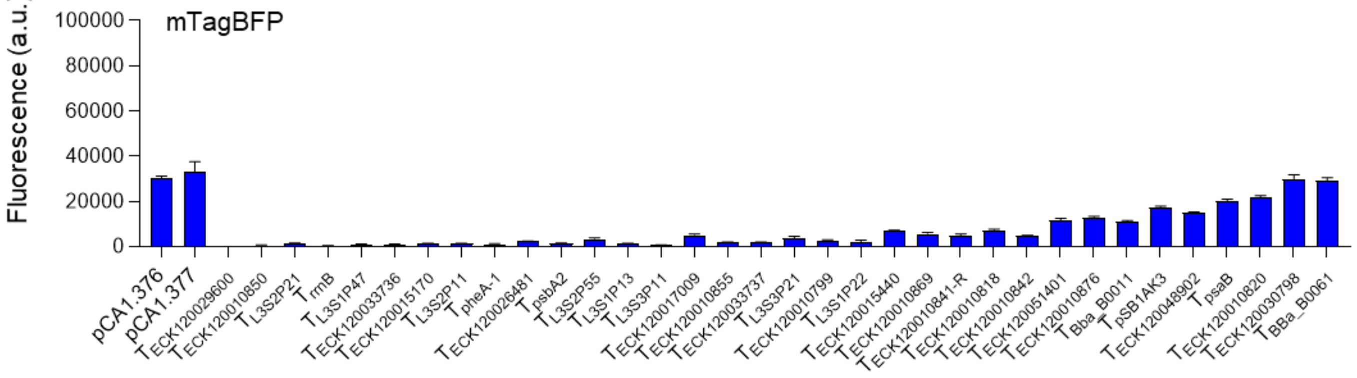

C

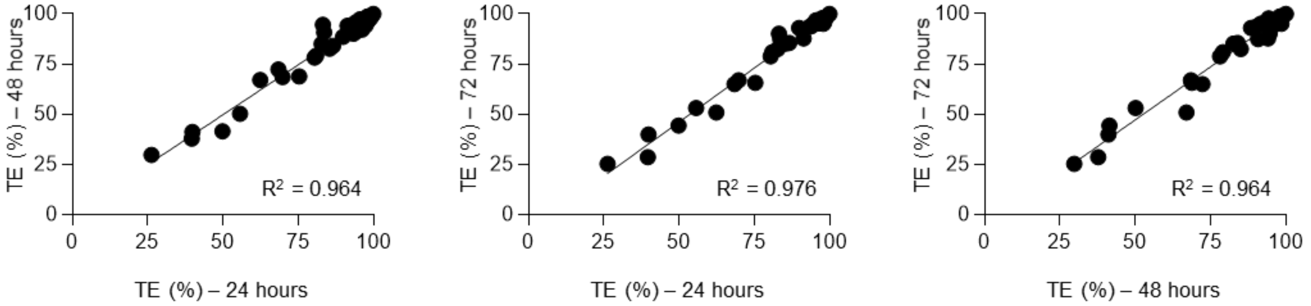

Supplementary Figure S4. Growth and fluorophore expression levels in *Synechococcus* UTEX 2973. **(A)** Cultures were grown for 72 hr at 40°C with continuous light (300 μmol photons m^{-2} s^{-1}). **(B)** Expression levels of eYFP and mTagBFP at three time points (24, 48 and 72 hr). Error bars represent the ±SE of the mean of four biological replicates, where each replicate represents the median measurement of at least 10,000 cells measured by flow cytometry. **(C)** Correlation analysis of TE values between the three different time points (n = 34). The coefficient of determination (R^2) is shown.

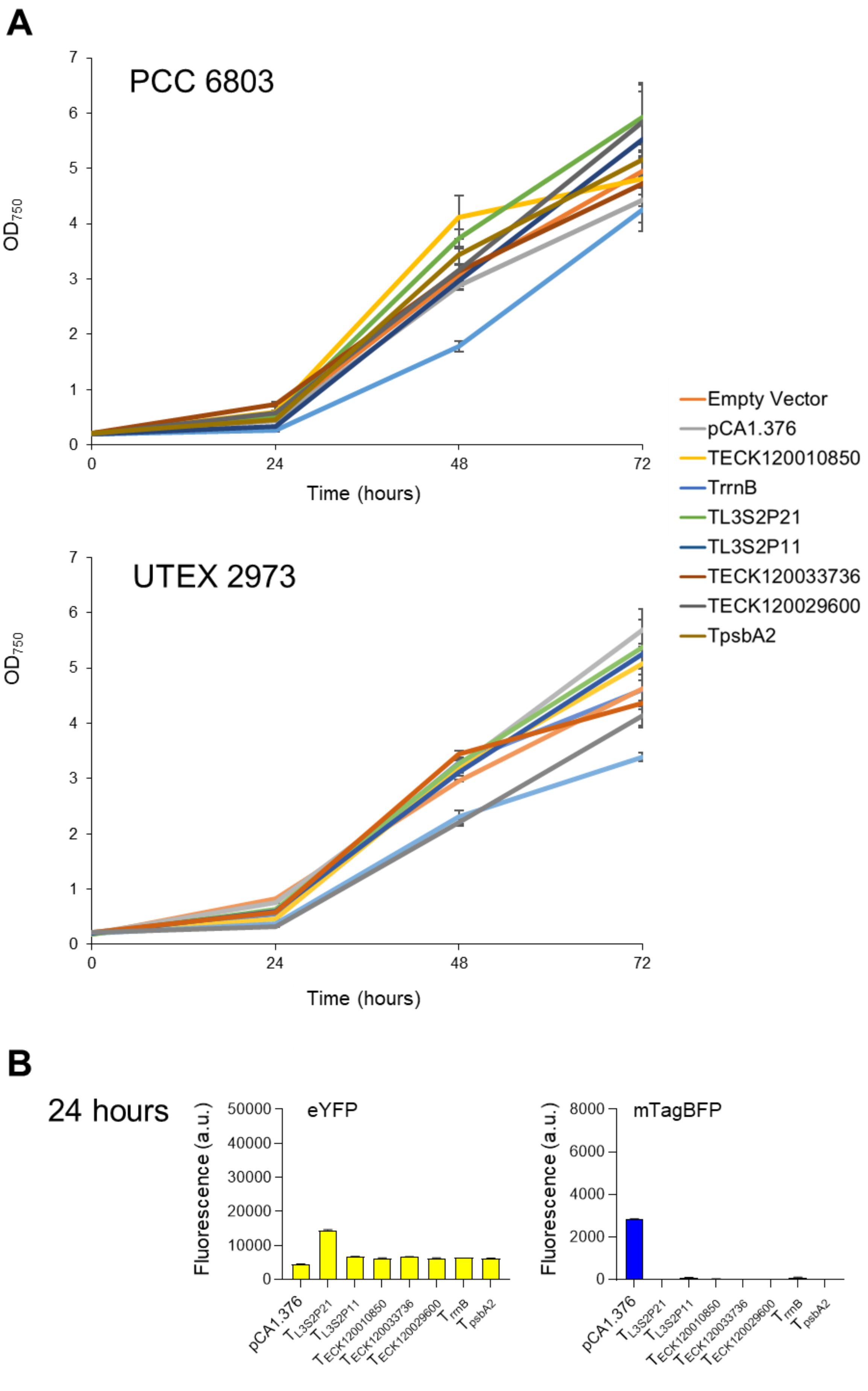
A
PCC 6803
UTEX 2973
OD750
Time (hours)
Empty Vector
pCA1.376
TECK120010850
TrrnB
TL3S2P21
TL3S2P11
TECK120033736
TECK120029600
TpsbA2
B
24 hours
eYFP
mTagBFP
Fluorescence (a.u.)
pCA1.376
TL3S2P21
TL3S2P11
TECK120010850
TECK120033736
TECK120029600
TrrnB
TpsbA2

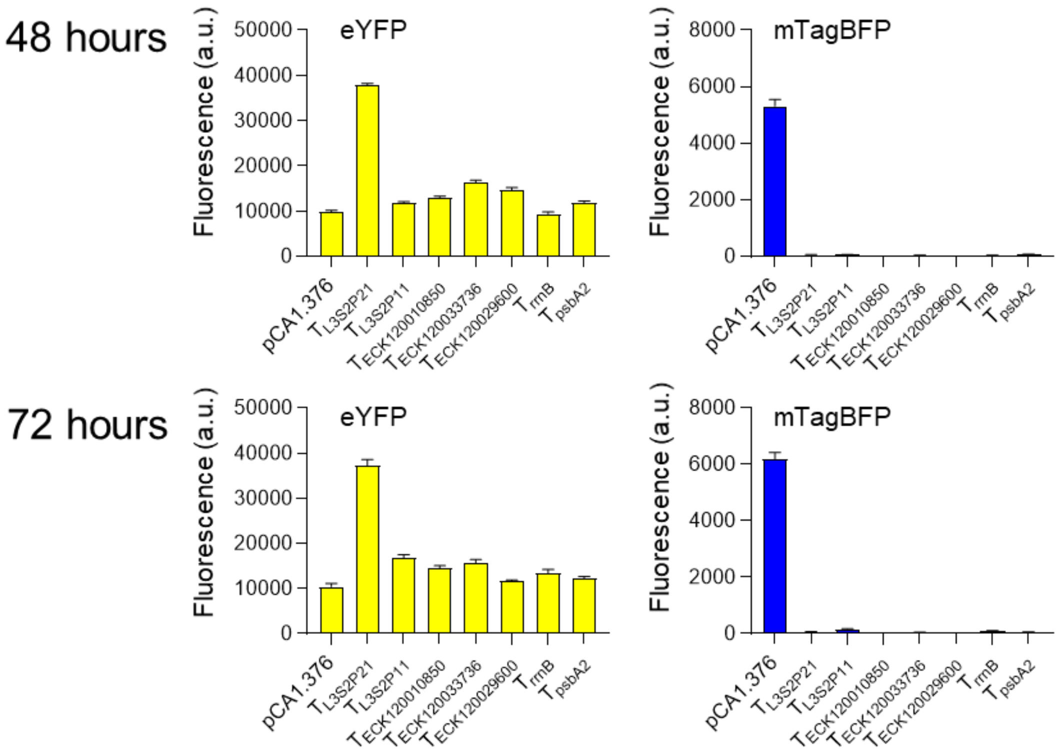
48 hours
eYFP
mTagBFP
72 hours
eYFP
mTagBFP
Fluorescence (a.u.)
pCA1.376
$T_{L3S2P21}$
$T_{L3S2P11}$
$T_{ECK120010850}$
$T_{ECK120033736}$
$T_{ECK120029600}$
T_{rrnB}
T_{psbA2}

C

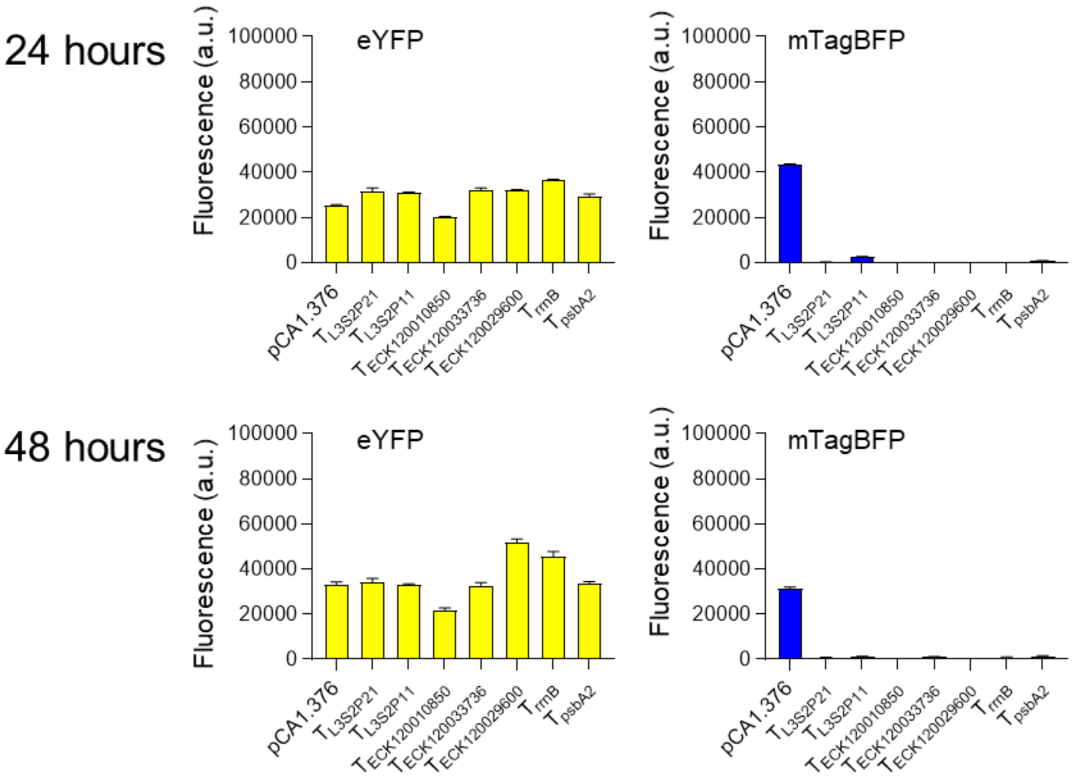
24 hours
eYFP
mTagBFP
48 hours
eYFP
mTagBFP
Fluorescence (a.u.)
pCA1.376
$T_{L3S2P21}$
$T_{L3S2P11}$
$T_{ECK120010850}$
$T_{ECK120033736}$
$T_{ECK120029600}$
T_{rrnB}
T_{psbA2}

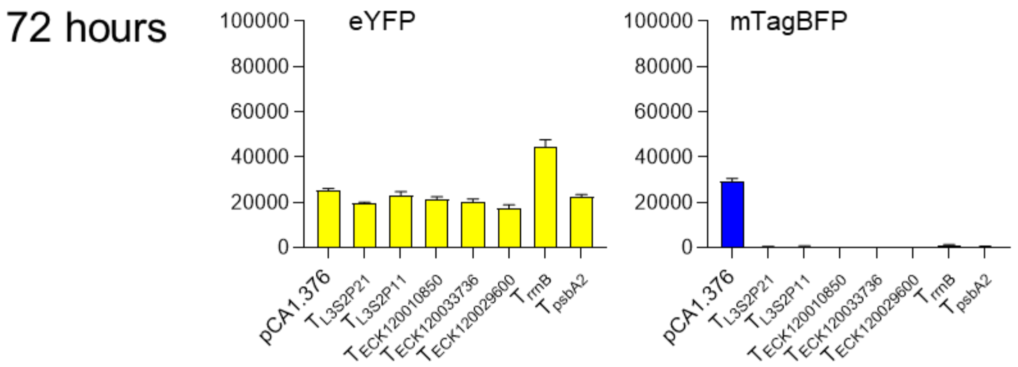

Supplementary Figure S5. Growth and fluorophore expression levels in *Synechocystis* sp. PCC 6803 and *Synechococcus* UTEX 2973 under suboptimal growth conditions. **(A)** Cultures were grown for 72 hr at 30°C with continuous high light (300 µmol photons m^{-2} s^{-1}). **(B)** Expression levels of eYFP and mTagBFP in PCC 6803 at three time points (24, 48 and 72 hr). **(C)** Expression levels of eYFP and mTagBFP in UTEX 2973 at three time points. Error bars represent the ±SE of the mean of four biological replicates, where each replicate represents the median measurement of at least 10,000 cells measured by flow cytometry.

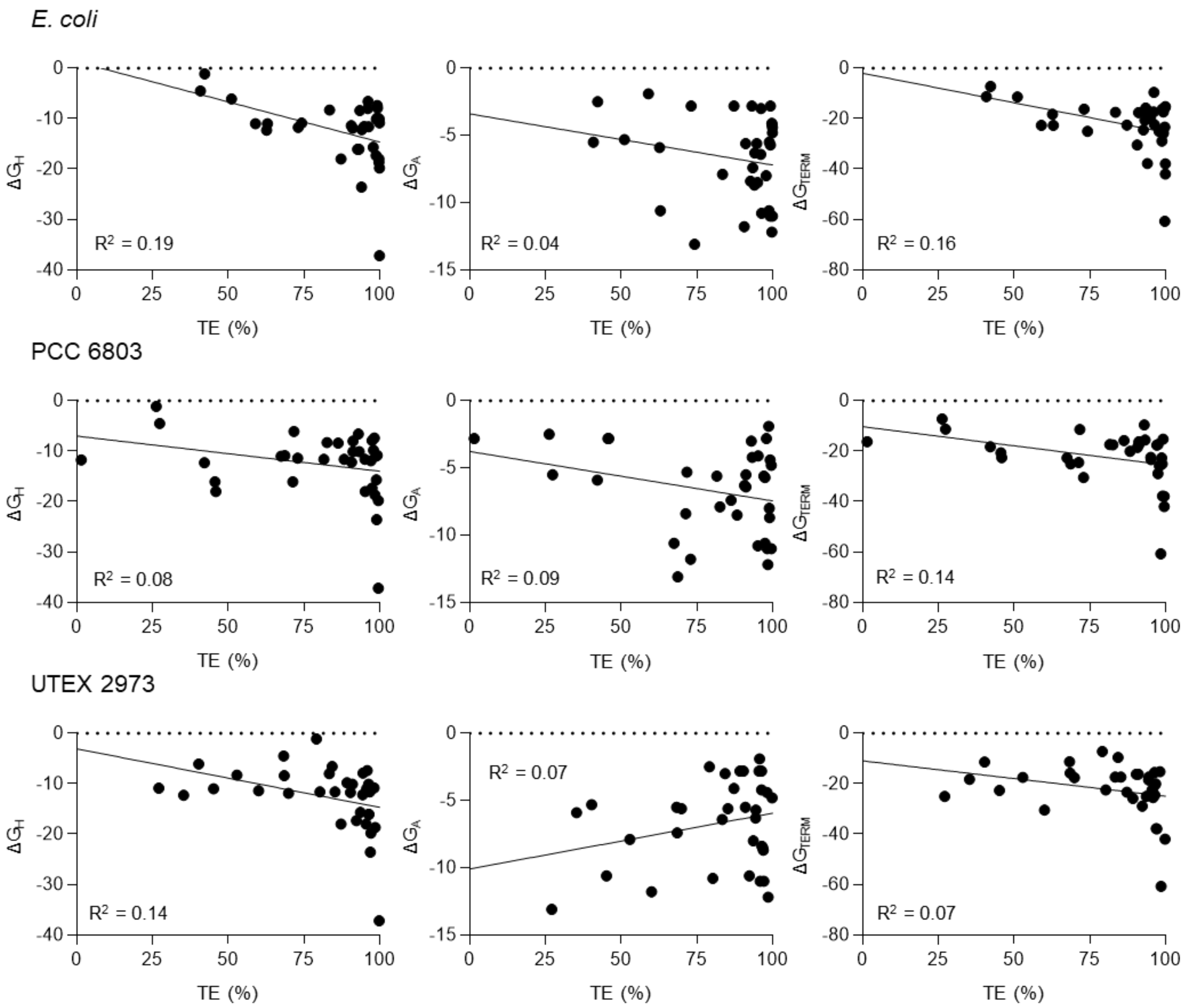

Supplementary Figure S6. Gibbs free energy values for terminator sequences plotted against TE values for *E. coli*, *Synechocystis* sp. PCC 6803 and *Synechococcus* UTEX 2973. Gibbs free energy values are taken from **Table 1** and **Supplementary Table S4**, TE values are from **Supplementary Table S3**.

```
slr0653              1 MTQTKEPL----------------TKAESAELEQEIELSQYINTDDIDDD
                       |||..|.|                .||.:|.:|....::...:.|.|||:
WP_071818124.1       1 MTQATELLDPALKPAETKAKRSSRKKATTAVVEPATTIAPTADVDAIDDE

slr0653             35 DIDVEDLEQEVAATEGKEKKVRKIRKDAVKKKPYTEDSIRIYLQEIGRIR
                       |...||   |.||.:.|.|          .:|.|||||||:|||||||||
WP_071818124.1      51 DSVGED---EDAAAKAKAK----------VRKTYTEDSIRLYLQEIGRIR

slr0653             85 LLRAEEEIELARQIADLLELELIRDNLTLQLERQPSELEWGKQVWKLETA
                       ||||:|||||||||||||.||.|||.|..||:|.||:.||...|
WP_071818124.1      88 LLRADEEIELARQIADLLALERIRDELLEQLDRLPSDAEWAAAV------

slr0653            135 KQRLVGDKKKEPKKKDIDSYLANPDNELSLENEWSQQPNKNFAAFRRRLF
                                        ||    |.:|                 ||||||
WP_071818124.1     132 -----------------DS----PLDE-----------------FRRRLF

slr0653            185 LDRRAKDKMVQSNLRLVVSIAKKYMNRGLSFQDLIQEGSLGLIRAAEKFD
                       ..||||||||||||||||||||||||||||||||||||||||||||||||
WP_071818124.1     144 RGRRAKDKMVQSNLRLVVSIAKKYMNRGLSFQDLIQEGSLGLIRAAEKFD

slr0653            235 HEKGYKFSTYATWWIRQAITRAIADQSRTIRLPVHLYETISRIKKTTKLL
                       ||||||||||||||||||||||||||||||||||||||||||||||||||
WP_071818124.1     194 HEKGYKFSTYATWWIRQAITRAIADQSRTIRLPVHLYETISRIKKTTKLL

slr0653            285 SQEMRRKPTEEEIAEKMEMTIEKLRFIAKSAQLPISLETPIGKEEDSRLG
                       ||||.|||||||||.:||||||||||||||||||||||||||||||||||
WP_071818124.1     244 SQEMGRKPTEEEIATRMEMTIEKLRFIAKSAQLPISLETPIGKEEDSRLG

slr0653            335 DFIEADGETPEDEVSKNLLREDLENVLDTLSPRERDVLRLRYGLDDGRMK
                       ||||||||||||||:|||||||||.||.||||||||||||||||||||||
WP_071818124.1     294 DFIEADGETPEDEVAKNLLREDLEGVLSTLSPRERDVLRLRYGLDDGRMK

slr0653            385 TLEEIGQIFNVTRERIRQIEAKALRKLRHPNRNSILKEYIR
                       |||||||:|||||||||||||||||||||||||||||||||
WP_071818124.1     344 TLEEIGQLFNVTRERIRQIEAKALRKLRHPNRNSILKEYIR
```

Supplementary Figure S7. Sequence alignment of SigA isoforms in *Synechocystis* sp. PCC 6803 and *Synechococcus* UTEX 2973. SigA from PCC 6803 (srl0653) and SigA1 from UTEX 2973 (WP_071818124.1) was aligned using EMBOSS Needle (https://www.ebi.ac.uk/Tools/psa/emboss_needle), which produced a shared identity of 70.5% and similarity of 74.1%. The amino acid residues shown in blue and red highlight the conserved regions required for recognition of the -10 and -35 boxes for prokaryotic promoters, respectively (Lonetto et al., 1992; Panaghie et al., 2000; Srivastava et al., 2020).

Supplementary References

Chen, Y. J., Liu, P., Nielsen, A. A. K., Brophy, J. A. N., Clancy, K., Peterson, T., & Voigt, C. A. (2013). Characterization of 582 natural and synthetic terminators and quantification of their design constraints. *Nature Methods*, *10*(7), 659–664. https://doi.org/10.1038/nmeth.2515

Lonetto, M., Gribskov, M., & Gross, C. A. (1992). The sigma 70 family: sequence conservation and evolutionary relationships. *Journal of Bacteriology*, *174*(12), 3843–3849. https://doi.org/10.1128/JB.174.12.3843-3849.1992

Panaghie, G., Aiyar, S. E., Bobb, K. L., Hayward, R. S., & De Haseth, P. L. (2000). Aromatic amino acids in region 2.3 of Escherichia coli sigma 70 participate collectively in the formation of an RNA polymerase-Promoter open complex. *Journal of Molecular Biology*, *299*(5), 1217–1230. https://doi.org/10.1006/jmbi.2000.3808

Srivastava, A., Varshney, R. K., & Shukla, P. (2020). Sigma Factor Modulation for Cyanobacterial Metabolic Engineering. *Trends in Microbiology*, 1–12. https://doi.org/10.1016/j.tim.2020.10.012
